# Resonance promoted ring-opening metathesis polymerization of twisted amides†
†Electronic supplementary information (ESI) available. See DOI: 10.1039/c9sc03602d


**DOI:** 10.1039/c9sc03602d

**Published:** 2019-08-30

**Authors:** Mizhi Xu, Krista K. Bullard, Aja M. Nicely, Will R. Gutekunst

**Affiliations:** a School of Chemistry and Biochemistry , Georgia Institute of Technology , 901 Atlantic Drive NW , Atlanta , Georgia 30332 , USA . Email: willgute@gatech.edu

## Abstract

The living ring-opening metathesis polymerization (ROMP) of an unsaturated twisted amide using the third-generation Grubbs initiator is described. Unlike prior examples of ROMP monomers that rely on angular or steric strain for propagation, this system is driven by resonance destabilization of the amide that arises from geometric constraints of the bicyclic framework. Upon ring-opening, the amide can rotate and rehybridize to give a stabilized and planar conjugated system that promotes living propagation. The absence of other strain elements in the twisted amide is supported by the inability of a carbon analogue of the monomer to polymerize and computational studies that find resonance destabilization accounts for 11.3 kcal mol^–1^ of the overall 12.0 kcal mol^–1^ ring strain. The twisted amide polymerization is capable of preparing high molecular weight polymers rapidly at room temperature, and post-polymerization modification combined with 2D NMR spectroscopy confirms a regioirregular polymer microstructure.

## Abstract 2

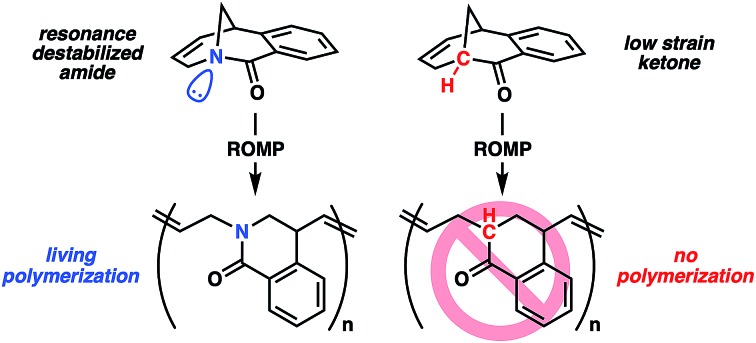
The twisting of an amide bond provides a new driving force for living ring-opening metathesis polymerization through resonance destabilization.

## Introduction

**Fig. 1:**
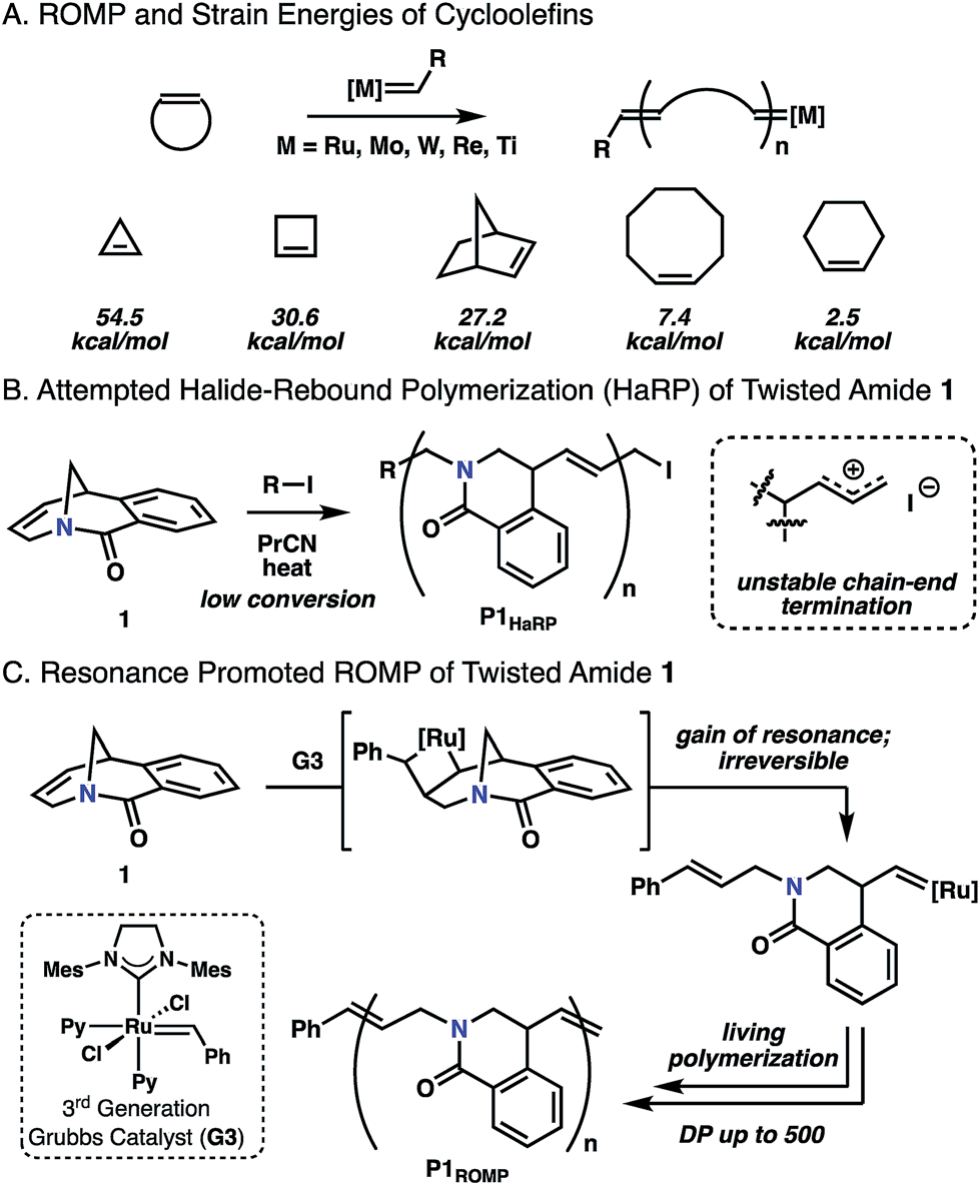
(A) Typical ROMP and cycloolefin monomers. (B) Halide-rebound polymerization of twisted amide **1**. (C) Resonance promoted ROMP of **1**.

Ring-strain is a common driving force for chemical reactions in both small molecule and macromolecular synthesis. This is the origin of remarkable carbon–carbon bond cleavage mechanisms of cyclopropane rings,^1^ as well as ring-opening polymerization of epoxides to give polyethers.^2^ Strained cycloolefins have been the monomer of choice in ring-opening metathesis polymerization (ROMP) where small and bicyclic cycloolefins have dominated the landscape.^3^ As olefin metathesis is fundamentally an exchange process, the key to successful polymerization is to shift the reaction equilibrium towards propagation. The strain energies of these systems have been documented by Schleyer,^4^ highlighting that the success of ROMP is directly related to the ring strain present in the monomer (Fig. 1A). Conventional consideration of energy in these systems is usually related to the angular or steric strain that arise from the cyclic geometry of the ring.^5^ Upon ring-opening, these bonds can relax to preferred geometries in an overall exothermic process. While clever methods have been explored to promote metathesis polymerization using entropic factors^6^ or tandem reactions,^7^ ring strain remains the dominant approach. In this report, a twisted amide monomer that lacks angular and steric strain is reported for ROMP in which living polymerization is enabled through resonance destabilization.

Twisted amides constitute an unusual class of molecules that have distorted, nonplanar amide N–C(O) bonds as a result of geometric, steric, or electronic effects.^8^ Since classical amides derive significant resonance stability from n_N_-to-π*_C=O_ orbital overlap, twisting hinders this interaction and results in nominal amides with unusual reactivity profiles. Hydrolytic stability and nitrogen basicity of these amides has been directly correlated to the degree of distortion, defined by the twist angle (*τ*) and nitrogen pyramidalization (*χ*_N_) parameters introduced by Winkler and Dunitz,^9^ as shown in a number of experimental and computational studies.^10^ Most of the early investigations in this area were physical organic in nature with the goal of understanding basic reactivity, though twisted amides are emerging as useful functional groups for a wide range of cross-coupling reactions^11^ and polymer post-functionalization.^12^

Recently, we applied the unconventional nucleophilicity of the amide nitrogen observed by Kirby,10c,10d Aubé10*n* and Szostak10g,10h to invent a covalent electrophilic polymerization of twisted amides termed halide-rebound polymerization (HaRP).^13^ In this process, the twisted amide monomers underwent alkylation of the amide nitrogen, followed by nucleophilic rebound of the halide counterion to generate isolable living polymers. In further studies, the unsaturated twisted amide **1** was explored in HaRP and only resulted in oligomers with broad molecular weight distributions (Fig. 1B and S5, S6†). The poor polymerization behaviour is proposed to be the result of ionization of the highly reactive allyl iodide chain-end at high temperature, leading to chain-end termination. While ineffective in HaRP, the double bond in amide **1** led to the consideration of ROMP for polymerization. The [3.3.1] bicyclic structure does not possess significant angular or steric strain, and Grubbs previously demonstrated that the [3.3.1] system readily forms through ring-closing metathesis in high yields at reasonable concentrations (0.05 M).^14^ While this would suggest the inability of twisted amide **1** to polymerize under ROMP conditions, the Winkler–Dunitz parameters (*τ* = 30.7°, *χ*_N_ = 49.7°) significantly deviate from planarity.10*h* The reduced n_N_-to-π*_C=O_ overlap causes the twisted amide to be higher in energy than a planar amide, and it was proposed that **1** would be competent in ROMP through the gain of resonance energy after ring-opening and planarization of the nitrogen atom to an sp^2^ hybrid (Fig. 1C). While other examples of ROMP have likely benefited in part from resonance effects, such as paracyclophene polymerization, these monomers have significant angular deformations that serve as the primary driving force.^15^ Herein, this concept is validated by the ROMP of twisted amide **1** using the 3rd generation Grubbs initiator (**G3**) (Fig. 1C).

## Results and discussion

**Fig. 2:**
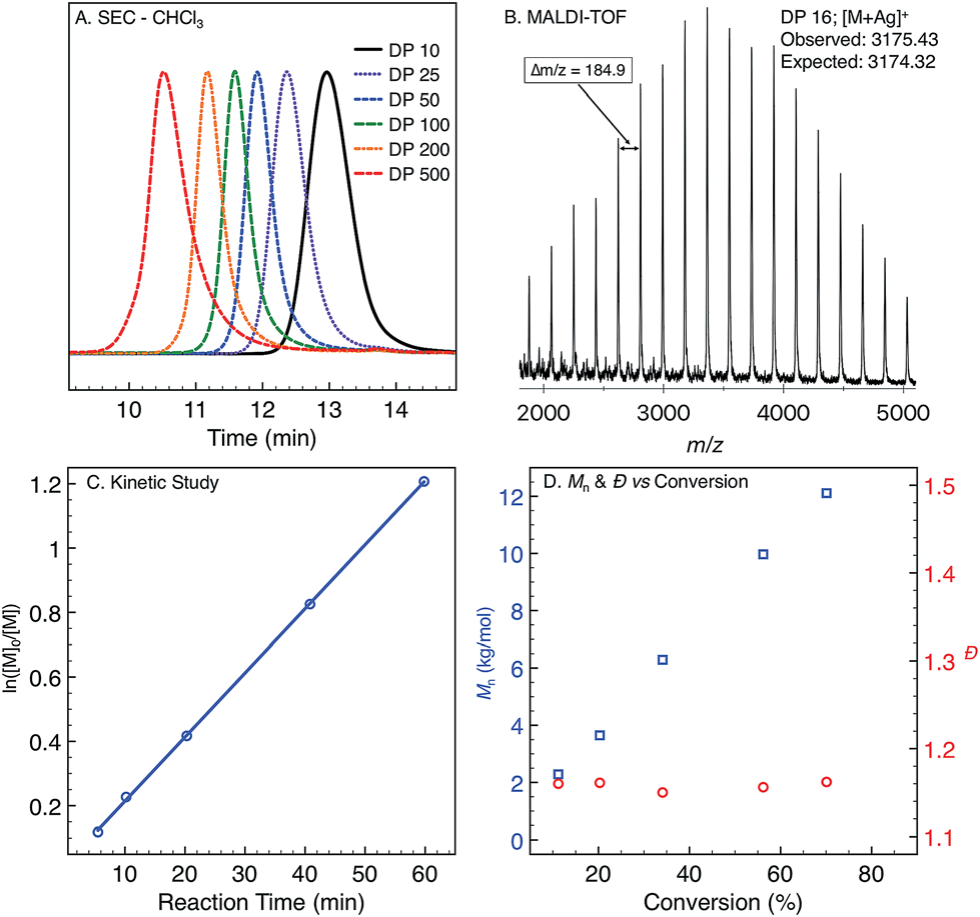
(A) Size-exclusion chromatograms of **P1_ROMP_** targeting different DPs. (B) MALDI-TOF-MS spectrum of **P1_ROMP_**. (C) First-order kinetic plot for **1** targeting DP 100. (D) *M*_n_-conversion correlation (blue) and *Ð*-conversion correlation (red).

Monomer **1** was prepared following the two-step protocol described by Grigg starting from 2-iodobenzoic acid and tetrahydropyridine.^16^ An initial ROMP experiment with monomer **1** was performed with **G3** in DCM at room temperature targeting a degree of polymerization (DP) of 100 (Table 1, entry 4). The polymerization reached 90% conversion in 2 hours and produced the polymer **P1_ROMP_** with a number-average molecular weight (*M*_n_) of 15.2 kg mol^–1^ and dispersity (*Ð*) of 1.20. By varying the monomer-initiator ratio, different molecular weights (DP = 10 to 500) were accurately targeted with high monomer conversions and unimodal elution peaks by size-exclusion chromatography (SEC) analysis (Table 1 and Fig. 2A). Higher molecular weight polymers had slightly increased dispersity values (entries 4 and 5, *Ð* = 1.26 and 1.46) implying some secondary metathesis of the backbone olefins occurred, which was supported by experiments at extended reaction times after full conversion was reached (Table S1 and Fig. S4†). The structure of **P1_ROMP_** was confirmed by MALDI-TOF-MS analysis (Fig. 2B). A single series of molecular ion peaks separated by 185 Da, the molecular mass of **1**, was observed and each peak was consistent with the calculated values for polymers containing phenyl and terminal olefin chain-ends. Kinetic analysis further reinforced the living nature of the twisted amide polymerization. Aliquots taken during an experiment targeting DP 100 were analysed for conversion and molecular weight by ^1^H NMR and SEC, respectively, to reveal first-order kinetic behaviour (Fig. 2C) and a linear increase in molecular weight with conversion (Fig. 2D). To better understand the kinetic behaviour, polymerizations of **1** were monitored using variable-temperature NMR experiments (Fig. S11 and Table S2†). The obtained rate constant (0.166 M^–1^ s^–1^ at 25 °C) is several orders of magnitude lower than the norbornene derivatives commonly used in ROMP (*e.g.* 63.2 M^–1^ s^–1^ at 25 °C for *exo*-butyl norbornene imide).^17^ An Eyring plot was obtained (Fig. S12†) based on the rate constants at different temperatures, showing enthalpy of activation Δ*H*^‡^ = 84.0 kJ mol^–1^, and entropy of activation Δ*S*^‡^ = –30.8 J mol^–1^ K^–1^.^18^ The negative entropy of activation suggests a possible intramolecular coordination of the propagating ruthenium alkylidene species by the neighbouring amide, though *in situ* NMR studies were unable to conclusively demonstrate the coordination state of the chain-end (Fig. S46 and S47†).^19^

**Table 1:** ROMP of twisted amide **1** targeting different degrees of polymerization (DPs)

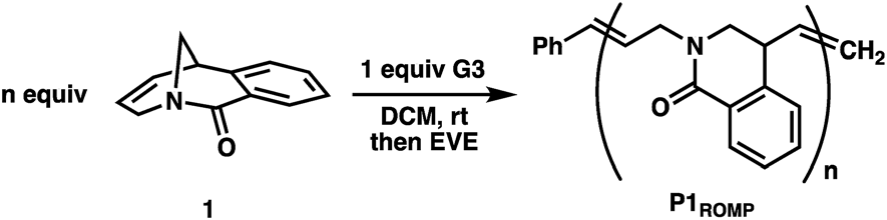						
Entry^*a*^	Target DP (*n*)	Time (h)	Conv.^*b*^ (%)	*M*_n,theor_ ^*c*^ (kg mol^–1^)	*M*_n,SEC_ ^*d*^ (kg mol^–1^)	*Ð* ^*d*^
1	10	2	95%	1.9	1.9	1.21
2	25	2	93%	4.4	4.6	1.20
3	50	2	91%	8.6	9.0	1.20
4	100	2	91%	16.9	15.2	1.20
5	200	2	89%	27.6	33.1	1.26
6	500	3	91%	84.7	63.4	1.46

^*a*^ROMP of **1** (0.3 mmol, 0.2 M) was initiated by **G3** in DCM at room temperature.

^*b*^Conversions were determined by ^1^H NMR of crude reaction mixture.

^*c*^ *M*_n,theo_ = *n* × conv. × M(**1**) + M(PhCH=CH_2_).

^*d*^Number average molecular weights (*M*_n_) and dispersities (*Ð*) of purified polymers were determined by size-exclusion chromatography using polystyrene standards.

**Fig. 3:**
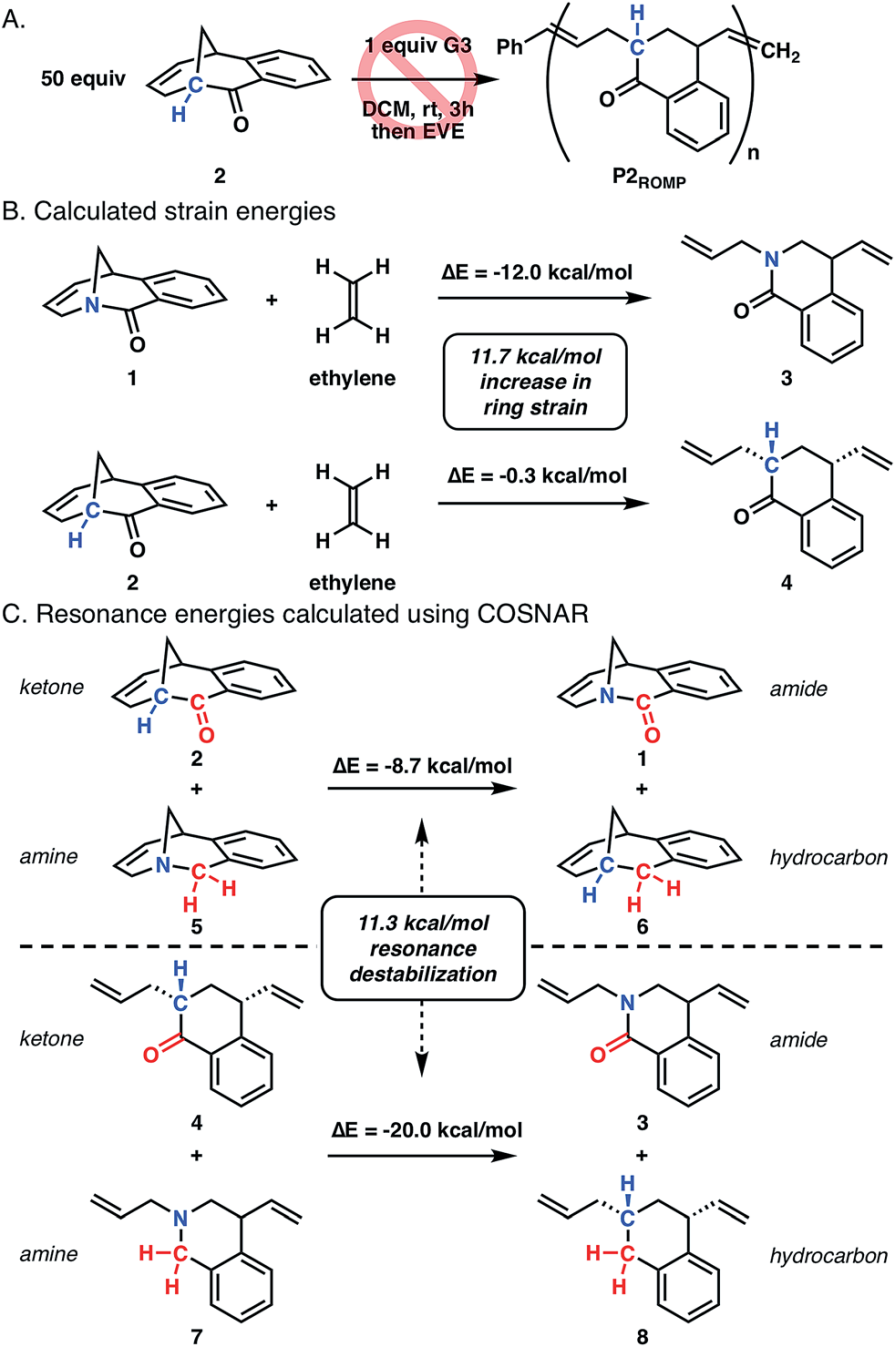
(A) Attempted ROMP of ketone **2**. (B) Calculated ring strain energies of twisted amide **1** and ketone **2** from isodesmic ring-opening reaction with ethylene. (C) Resonance energies of amide **1** and **3** determined by COSNAR method (B3LYP-D3MBJ/6-311++G(d,p)).

To better understand the role of amide distortion in the polymerization, a ROMP experiment was performed with the ketone analogue **2** in which the bridgehead nitrogen of twisted amide **1** is replaced with a methine carbon (Fig. 3A). This molecule provides an ideal evaluation of the impact of the amide functionality, though the carbon substitution would lead to slightly altered ground state geometries due to differences in C–C and C–N bond lengths and hybridization states. Ketone **2** was synthesized in three steps from 1,2-diiodobenzene using a similar Heck-strategy reported for the synthesis of twisted amide **1** (Scheme S2†). Upon subjection of **2** to the polymerization conditions developed for twisted amide ROMP (0.02 equiv. **G3**, 0.2 M in DCM), no reaction was observed after three hours at room temperature (Fig. 3A) which is consistent with facile formation of the [3.3.1] bicyclic structure observed by Grubbs.^14^

Computational studies were performed at the B3LYP-D3MBJ/6-311++G(d,p) level of theory to gain more insight into the differences in ring strain between monomers **1** and **2**, which has been shown to be suitable for twisted amide calculations.10o–10s,^20^ The ring strain of twisted amide **1** was found to be 12.0 kcal mol^–1^ using an isodesmic reaction for the ring-opening with ethylene (Fig. 3B).^21^ Analogous calculations with **2** and ethylene showed a very low ring strain for the ketone (0.3 kcal mol^–1^) that is consistent with inability of **2** to polymerize. To separate the contribution of resonance destabilization from the overall ring strain in **1**, the carbonyl substitution nitrogen atom replacement (COSNAR) method developed by Greenberg was applied.10r,10s This isodesmic reaction determines the resonance energy of an amide by comparing the overall energies of amine/ketone and hydrocarbon/amide combinations with the same structural framework. The resonance energies of twisted amide **1** and ring-opened amide **3** were calculated to be 8.7 kcal mol^–1^ and 20.0 kcal mol^–1^, respectively (Fig. 3C). The 11.3 kcal mol^–1^ difference between these two energies represents the contribution of resonance destabilization to the total ring strain in the system and is very similar to the difference in ring strain between **1** and **2** (11.7 kcal mol^–1^). This indicates that resonance destabilization is responsible for over 90% of the ring strain in **1** (12.0 kcal mol^–1^), further supporting gain of resonance energy as the primary driving force for polymerization.

**Fig. 4:**
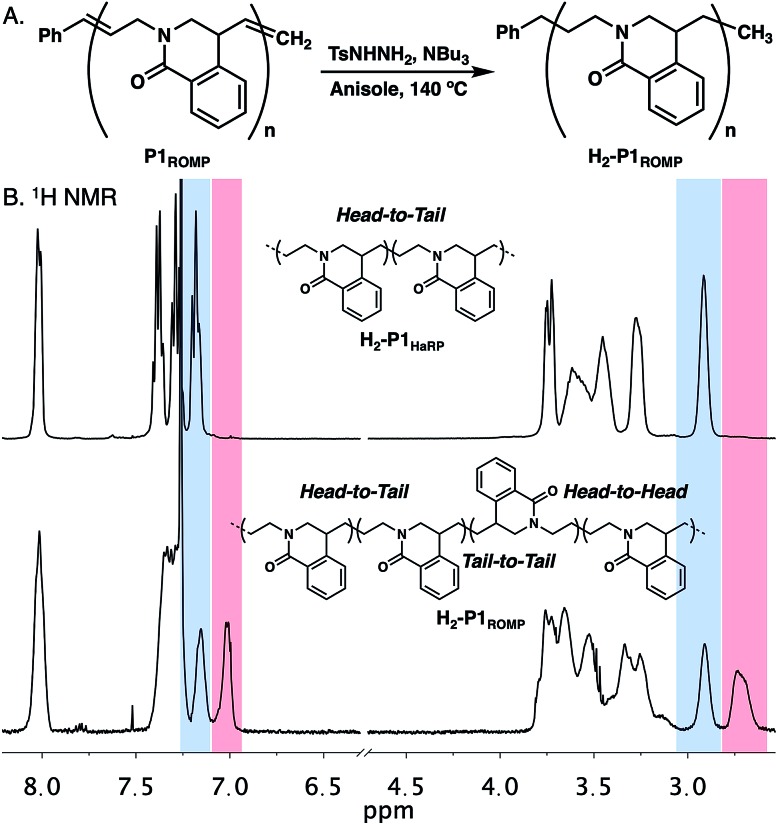
(A). Reduction of **P1_ROMP_** to generate saturated polymer **H_2_-P1_ROMP_**. (B) Stacked ^1^H NMR spectra and microstructures of **H_2_-P1_HaRP_** and **H_2_-P1_ROMP_**.

The polymerization of amide **1** through HaRP and ROMP should give the same polymer structure. While only oligomers formed under HaRP conditions, slight differences were noted in the ^1^H NMR spectra of **P1_HaRP_** and **P1_ROMP_** (Fig. S7†). It was not possible to determine by ^1^H or ^13^C NMR experiments if this originated from differences in olefin *E*/*Z* isomer populations or a change in polymer regioregularity. Since the olefin in twisted amide **1** is asymmetric, ring-opening in either direction would provide two different propagating species leading to head-to-tail (HT), head-to-head (HH) or tail-to-tail (TT) connectivity (Fig. S16†). To remove the complications of olefin geometry, **P1_ROMP_** was hydrogenated with diimide to give saturated **H_2_-P1_ROMP_** for comparison with the analogous polymer prepared through HaRP (**H_2_-P1_HaRP_**, Fig. 4A).^22^ The mechanism of HaRP is inherently regioregular, facilitating the assignment of the ROMP microstructure. A stacked comparison of ^1^H NMR spectra between **H_2_-P1_ROMP_** and **H_2_-P1_HaRP_** shows common signals between the two polymers (blue highlight), unique signals at 7.0 ppm and 2.7 ppm for **H_2_-P1_ROMP_** (red highlight), as well as clear differences in the 3.1–3.9 ppm and 7.3–7.5 ppm regions (Fig. 4B). Further 2D NMR analysis showed that the highlighted peaks of **P3_ROMP_** (blue & red) correlated to head-to-tail and tail-to-tail connectivity at the benzylic position, implying indiscriminate ring-opening of the monomer from either side of the olefin (Fig. S17†). The relative integration of the isolated protons to the common protons at 8.0 ppm further supports the lack of preference in the ring-opening step and establishes the regiorandom nature of the polymers. Inspection of the crystal structure of **1** reported by Szostak showed limited steric bias in the approach of a propagating alkylidene to either side of the twisted amide double bond in agreement with these results (Fig. S15†).

Given the different oxidation states and microstructures of the twisted amide polymers prepared through ROMP and HaRP, thermal analysis was performed to determine further distinctions between these isomeric materials. Thermogravimetric analysis (TGA) showed slightly lower thermal stability of **P1_ROMP_** compared to **H_2_-P1_HaRP_** (10% mass loss at 368 °C *versus* 401 °C, Fig. S18†) likely due to the lower bond dissociation energies of the head-to-head or tail-to-tail linkages.^13^ Differential scanning calorimetry (DSC) experiments displayed a higher glass transition temperature of **P1_ROMP_** than **H_2_-P1_HaRP_** (158 °C *versus* 123 °C, Fig. S19†), which is consistent with a more rigid olefinic backbone. A glass transition temperature of **H_2_-P1_ROMP_** was observed at 120 °C (Fig. S19†), indicating the regioregularity of saturated polymer chains has limited effect on the glass transition.

## Conclusions

The ring-opening metathesis polymerization of the unsaturated twisted amide monomer **1** has been described that lacks the angular and steric strain elements traditionally found in ROMP monomers. In contrast, this system leverages resonance destabilization of an amide bond to promote living polymerization to high molecular weights. This is supported by the inability of the bicyclic ketone analogue **2** to polymerize under standard ROMP conditions and computational experiments that highlight the central role of resonance destabilization in the overall monomer ring strain. The microstructure of the resultant polymer **P1_ROMP_** was determined to be regioirregular through hydrogenation experiments coupled to 2D NMR analysis. The monomer orientation had a minimal effect on the glass transition temperature of the polymer but was found to lower overall thermal stability. Future work is underway to design new unsaturated twisted amide structures for ROMP that are promoted by resonance destabilization, as well as exploring applications of this new materials class.

## Conflicts of interest

There are no conflicts to declare.

## Supplementary Material

Supplementary information
