# Supplementary material for: Resonance promoted ring-opening metathesis polymerization of twisted amides
Source: Chem Sci. 2019 Aug 30;10(42):9729–34. doi: 10.1039/c9sc03602d (PMC6993617; doi:10.1039/c9sc03602d)
Supplement: Supplementary file 1 [file SC-010-C9SC03602D-s001.pdf]

Electronic Supplementary Material (ESI) for Chemical Science.
This journal is © The Royal Society of Chemistry 2019

Electronic Supplementary Information

Resonance Promoted Ring-Opening Metathesis Polymerization of Twisted Amides

Mizhi Xu, Krista K. Bullard, Aja M. Nicely and Will R. Gutekunst*

School of Chemistry and Biochemistry, Georgia Institute of Technology,
901 Atlantic Drive NW, Atlanta,
Georgia 30332, United States
willgute@gatech.edu

Table of Contents

General Methods ---2
Experimental Procedures ---2-15
General Ring-Opening Metathesis Polymerization (ROMP) Procedure/Examples ---------------------------4-6
Halide-Rebound Polymerization (HaRP) of **1**---6-8
Attempted Polymerization of Ketone **2** --8-9
Polymerization Kinetic Studies ---9-11
Regioregularity Studies --12-14
Polymer Thermal Properties --15
Computational Studies ---16-22
^{1}H, ^{13}C and 2D NMR Spectra --23-34
References ---34

General Methods

All reactions were carried out under a nitrogen atmosphere with dry solvents using anhydrous conditions unless otherwise stated. Dry, degassed dichloromethane (DCM), acetonitrile (CH_3CN), *N,N*-dimethylformamide (DMF) and tetrahydrofuran (THF) were obtained from a JC Meyer solvent purification system. 3rd Generation Grubbs catalyst (**G3**) was prepared according to a literature method.[1] Unless otherwise stated, all other reagents were purchased at the highest commercial quality and used without further purification. Yields refer to chromatographically and spectroscopically (^{1}H-NMR) homogeneous materials, unless otherwise stated. Reactions were monitored by thin layer chromatography (TLC) carried out on 0.25 mm E. Merck silica gel plates (60F-254) using UV light as the visualizing agent and basic aqueous potassium permanganate ($KMnO_4$), and heat as developing agents. Silica gel (60 Å, 40-63 μm (230-400 mesh)) from Silicycle was used for flash column chromatography. NMR spectra were recorded on Bruker Avance 400 or 500 MHz instruments and calibrated using residual undeuterated solvent as an internal reference ($CHCl_3$ @ 7.26 ppm ^{1}H NMR, 77.16 ppm ^{13}C NMR), and analyzed by MestReNova (version: 11.0.4). The following abbreviations (or combinations thereof) were used to explain the multiplicities: s = singlet, d = doublet, t = triplet, q = quartet, m = multiplet, br = broad. Mass spectra (MS) were recorded on GC/MS (Agilent Technologies 7890A GC system/5975C with Triple-Axis Detector) or time-of-flight matrix assisted laser desorption/ionization (MALDI-TOF) using a *trans*-2-[3-(4-tert-butylphenyl)-2-methyl-2-propenylidene]malononitrile (DCTB) matrix. Polymer samples were analyzed using a Tosoh EcoSEC HLC 8320GPC system with TSKgel SuperHZ-L columns eluting $CHCl_3$ containing 0.25% NEt_3 at a flow rate of 0.45 mL/min. All number-average molecular weights and dispersities were calculated from refractive index chromatograms using PStQuick Mp-M polystyrene standards. Thermogravimetric analyses (TGA) were performed under nitrogen atmosphere on TGA/DSC 3^{+} STAR^{e} system (Mettler Toledo) at a heating rate of 10 °C/min. Differential scanning calorimetry (DSC) analyses were measured on a DSC 3^{+} STAR^{e} system (Mettler Toledo) under nitrogen atmosphere, and the reported data were obtained from the second or third heating cycle at a heating rate of 10 °C/min.

Experimental Procedures

Scheme S1. Synthesis of Twisted Amide 1.

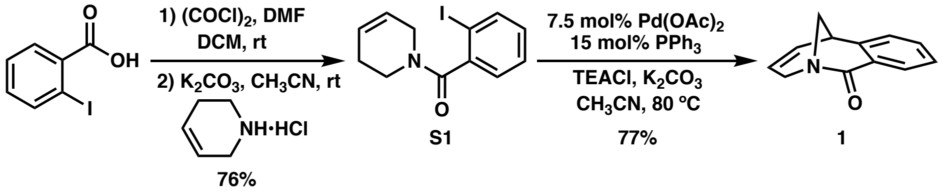

Twisted amide **1** was prepared according to a modified literature method.[2]

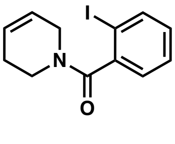

S1: A 500 mL oven-dried round-bottom flask equipped with a stir bar was charged with 2-iodobenzoic acid (12.40 g, 50 mmol, 1 equiv) and DCM (200 mL). Oxalyl chloride (7.62 g, 5.15 mL, 60 mmol, 1.2 equiv) was slowly added to the mixture, followed by addition of DMF (0.2 mL). The reaction mixture was stirred overnight at room temperature. Then solvent and excess oxalyl chloride were removed *in vacuo*. 1,2,3,6-tetrahydropyridine hydrochloride (5.68 g, 47.5 mmol, 0.95 equiv), potassium carbonate (17.28 g, 125 mmol, 2.5 equiv) and acetonitrile (150 mL) were added, and the reaction mixture was stirred overnight at room temperature. The solvent was removed *in vacuo*, and the residue was purified by column chromatography (10% to 33% EtOAc in hexanes) to give **S1** as a colorless oil (11.32 g, 76%). The ^{1}H and ^{13}C NMR data matched the data from literature.[2b]

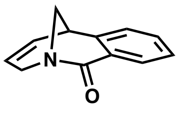

1: A 500 mL oven-dried round-bottom flask equipped with a stir bar was charged with amide **S1** (11.32 g, 36.15 mmol, 1 equiv), palladium acetate (0.61 g, 2.71 mmol, 0.075 equiv), triphenylphosphine (1.42 g, 5.42 mmol, 0.15 equiv), tetraethylammonium chloride (5.99 g, 36.15 mmol, 1 equiv), potassium carbonate (9.99 g, 72.30 mmol, 2 equiv) and acetonitrile (180 mL). The reactions were stirred at 80 °C under nitrogen atmosphere overnight, then cooled to room temperature. The mixture was passed through Celite and the filtrate was concentrated. The residue was purified by column chromatography (10% to 20% EtOAc in hexanes) to give **1** as a white solid (5.14 g, 77%). The ^{1}H and ^{13}C NMR data matched the data from literature.[2a,2b]

Scheme S2. Synthesis of Ketone 2.

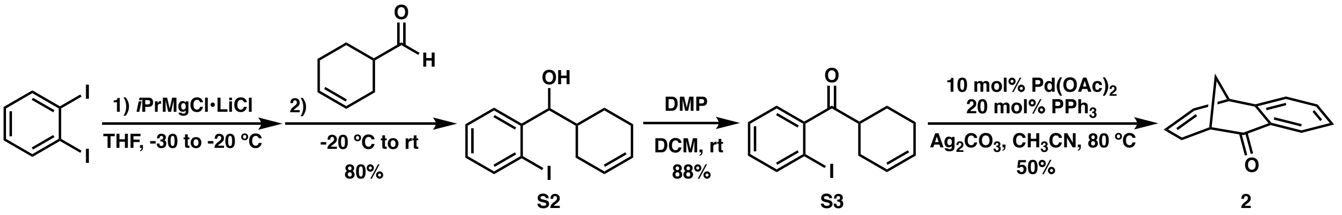

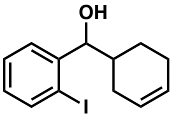

S2 was prepared using a modified literature method.[3] In a Schlenk flask, 1,2-diiodobenzene (1.98 g, 0.79 mL, 6 mmol, 1.2 equiv) was dissolved in anhydrous THF (18 mL). After cooling to -30 °C (cooling bath: ethylene glycol/ethanol=2/1, v/v, in dry ice), isopropylmagnesium chloride lithium chloride (Turbo Grignard reagent, 6 mmol, 1.3 M solution in THF, 4.61 mL) was added dropwise and the resulting orange colored mixture was warmed to -20 °C over a period of 20 min. The reaction was monitored by GC/MS.

After the formation of the *ortho*-iodophenyl Grignard reagent *in situ*, a solution of cyclohex-3-ene-1-carbaldehyde (551 mg, 5 mmol, 1 equiv) in THF (6 mL) was added dropwise at -20 °C and the mixture was allowed to warm up to room temperature overnight. The reaction was then diluted with Et_2O (10 mL), hydrolyzed in an ice bath with aqueous sat. NH_4Cl (30 mL). The layers were separated, and the aqueous phase was extracted with Et_2O (2 x 30 mL). The combined organic layers were dried (Na_2SO_4) and concentrated. The residue was purified by column chromatography (5% to 20% EtOAc in hexanes) to give **S2** as a colorless oil (1.26 g, 80%). ^{1}H NMR (400 MHz, $CDCl_3$, 25 °C) (a mixture of diastereomers): δ = 7.82 (dt, *J* = 7.9, 1.3 Hz, 1H), 7.46 (ddd, *J* = 7.8, 4.1, 1.8 Hz, 1H), 7.37 (tt, *J* = 7.5, 1.3 Hz, 1H), 6.97 (ddt, *J* = 9.0, 7.3, 1.6 Hz, 1H), 5.74-5.56 (m, 2H), 4.80 (dd, *J* = 17.4, 5.5 Hz, 1H), 2.19-1.70 (m, 7H), 1.63-1.38 (m, 1H); ^{13}C NMR (101 MHz, $CDCl_3$, 25 °C) (a mixture of diastereomers) δ = 145.4, 145.4, 139.5, 139.5, 129.3, 129.3, 128.5, 128.5, 128.2, 128.1, 127.2, 126.9, 126.4, 126.4, 99.0, 98.9, 81.3, 80.6, 40.3, 40.1, 28.5, 26.5, 25.9, 25.6, 25.2, 23.4; GC-MS (*m/z*): calcd for $C_{13}H_{15}IO$, $[M]^+$, 314.0; found, $[M-H_2O]^+$, 296.0.

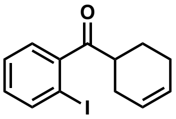

S3: To a solution of **S2** (942 mg, 3 mmol, 1 equiv) in wet DCM was added Dess-Martin periodinane (DMP, 2.54 g, 6 mmol, 2 equiv), and the reaction mixture was stirred at room temperature for 3h. The mixture was diluted with DCM and washed with a 1:1 mixture of 10% $Na_2S_2O_3$ and aqueous sat. $NaHCO_3$ (30 mL x 2), followed by water (30 mL) and brine (30 mL). The combined organic layers were dried (Na_2SO_4) and concentrated. The residue was purified by column chromatography (5% to 10% EtOAc in hexanes) to give **S3** as a colorless oil (833 mg, 88%). ^{1}H NMR (400 MHz, $CDCl_3$, 25 °C): δ = 7.90 (dd, *J* = 7.9, 1.1 Hz, 1H), 7.40 (td, *J* = 7.5, 1.1 Hz, 1H), 7.28 (dd, *J* = 7.6, 1.7 Hz, 1H), 7.12 (td, *J* = 7.7, 1.7 Hz, 1H), 5.77-5.67 (m, 2H), 3.29 (dddd, *J* = 11.3, 10.1, 5.4, 2.8 Hz, 1H), 2.42-1.99 (m, 5H), 1.75-1.62 (m, 1H); ^{13}C NMR (101 MHz, $CDCl_3$, 25 °C) δ = 208.1, 145.2, 140.3, 131.3, 128.0, 127.7, 126.8, 125.5, 91.4, 45.5, 27.1, 24.9, 24.8; GC-MS (*m/z*): calcd for $C_{13}H_{13}IO$, $[M]^+$, 312.0; found, $[M]^+$, 312.0.

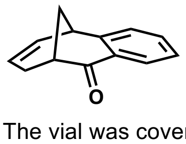

2 was synthesized using a modified literature method.[4] To an 8 mL vial equipped with a stir bar were added **S3** (62.4 mg, 0.2 mmol, 1 equiv), palladium acetate (4.5 mg, 0.02 mmol, 0.1 equiv), triphenylphosphine (10.5 mg, 0.04 mmol, 0.2 equiv), silver carbonate (110.3 mg, 0.4 mmol, 2 equiv), and acetonitrile (2 mL) at room temperature. The vial was covered by aluminum foil and heated at 80 ºC under N_2 for two days, then cooled to room temperature. The mixture was passed through Celite and the filtrate was concentrated. The residue was purified by column chromatography (2% to 8% Et_2O in hexanes) to give **3** as a volatile colorless oil (18.5 mg, 50%). ^{1}H NMR (500 MHz, $CDCl_3$, 25 °C): δ = 8.03 (dd, *J* = 7.8, 1.4 Hz, 1H), 7.43 (td, *J* = 7.4, 1.4 Hz, 1H), 7.28 (td, *J* = 7.6, 1.3 Hz, 1H), 7.23 (d, *J* = 7.5 Hz, 1H), 6.00 (dddt, *J* = 9.3, 6.1, 2.9, 1.5 Hz, 1H), 5.56 (ddd, *J* = 9.8, 4.6, 2.5 Hz, 1H), 3.39-3.34 (m, 1H), 3.00 (ddd, *J* = 7.2, 3.8, 1.9 Hz, 1H), 2.58 (ddtd, *J* = 18.8, 7.0, 2.7, 1.3 Hz, 1H), 2.44 (dddt, *J* = 12.5, 4.0, 2.7, 1.4 Hz, 1H), 2.26 (ddq, *J* = 18.8, 4.5, 1.5 Hz, 1H), 2.21 (ddd, *J* = 12.5, 3.4, 2.1 Hz, 1H); ^{13}C NMR (126 MHz, $CDCl_3$, 25 °C): δ = 202.4, 150.5, 133.6, 132.2, 131.5, 128.5, 126.7, 126.7, 124.9, 41.1, 35.6, 30.7, 29.7; 2D NMR experiments confirm the

position of the alkene double bond (COSY, HSQC and HMBC, see Figure S33-S35); GC-MS (*m/z*): calcd for $C_{13}H_{12}O$, $[M]^+$, 184.1; found, $[M]^+$, 184.1.

General Ring-Opening Metathesis Polymerization (ROMP) Procedure/Examples

Twisted amide **1** was dissolved in DCM in a 4 mL vial, followed by rapid addition of a **G3** stock solution in DCM to form reaction solution (0.2 M for the monomer). The reaction was stirred at room temperature for indicated time, and then quenched by ethyl vinyl ether (EVE) and the solvent was removed under vacuum. A small amount of the crude product was dissolved in $CDCl_3$ to determine the monomer conversion by ^1H NMR. The product was purified by precipitating into Et_2O/hexane (1/1, v/v), and then characterized using SEC, NMR and MALDI-TOF-MS analyses.

ROMP Condition Screening

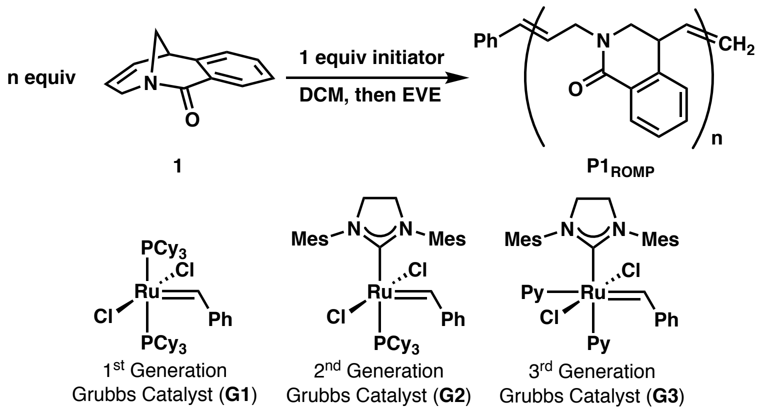

Table S1 Reaction condition screening for ROMP of **1**

Entry	Initiator	Temperature	Time	Target DP (n)	Monomer Concentration	Conversion[a]	M_n (kg/mol)[b]	*Đ*[b]
1	**G3**	rt	2 h	100	0.2 M	90%	19.0	1.21
2	**G3**	rt	17 h	100	0.2 M	>99%	20.7	1.37
3	**G3**	rt	70 h	100	0.2 M	>99%	17.8	1.54
4	**G3**	0 °C	3 h	50	0.2 M	17%	1.5	1.15
5	**G3**	rt	3.5 h	500	0.2 M	88%	66.5	1.49
6	**G3**	rt	3 h	500	0.4 M	88%	81.8	1.56
7	**G3**	rt	3 h	500	1.0 M	95%	79.6	1.58
8[c]	**G1**	50 °C	overnight	100	0.2 M	N.R.	N.A.	N.A.
9	**G2**	rt	3 h	100	0.2 M	>99%	33.6	2.66

[a] Conversions were determined by ^1H NMR of crude reaction mixture. [b] Number average molecular weights and dispersities of crude polymers were determined by size-exclusion chromatography using polystyrene standards. [c] Reaction in chloroform.

Example: ROMP of 1 (Targeting DP 100)

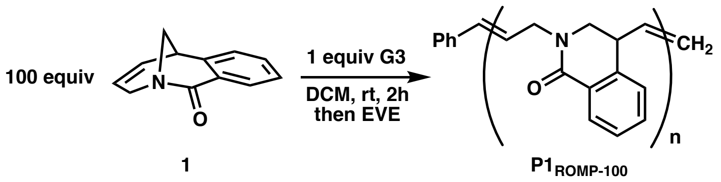

Twisted amide **1** (55.57 mg, 0.3 mmol, 100 equiv) was dissolved in DCM (1400 μL) in a 4 mL vial. A solution of **G3** (2.18 mg, 0.003 mmol, 1 equiv) in DCM (100 μL) was rapidly added to the twisted amide solution with stirring. After stirring at room temperature for 2 h,

the reaction was quenched by EVE (100 µL) and the solvent was removed under vacuum. A small amount of the crude product was dissolved in $CDCl_3$ to determine the monomer conversion by ^1H NMR (91%). The crude polymer **P1$_{ROMP-100}$** was precipitated three times by slowly adding a concentrated DCM solution (2 mL) into a Et_2O/hexane mixture (9 mL/9 mL) with vigorous stirring, and then dried under vacuum. The purified polymer was then characterized using ^1H-NMR and SEC analyses (M_n: 15.2 kg/mol, $Đ$: 1.20).

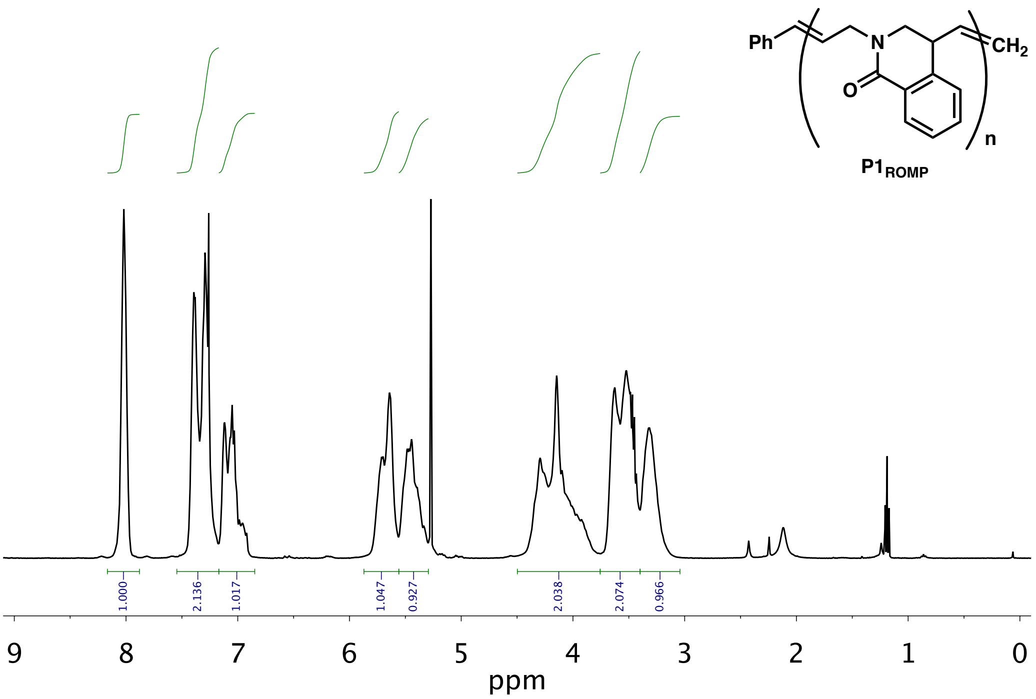

Figure S1. ^1H NMR (400 MHz, $CDCl_3$, 25 °C) of polymer **P1$_{ROMP-100}$**.

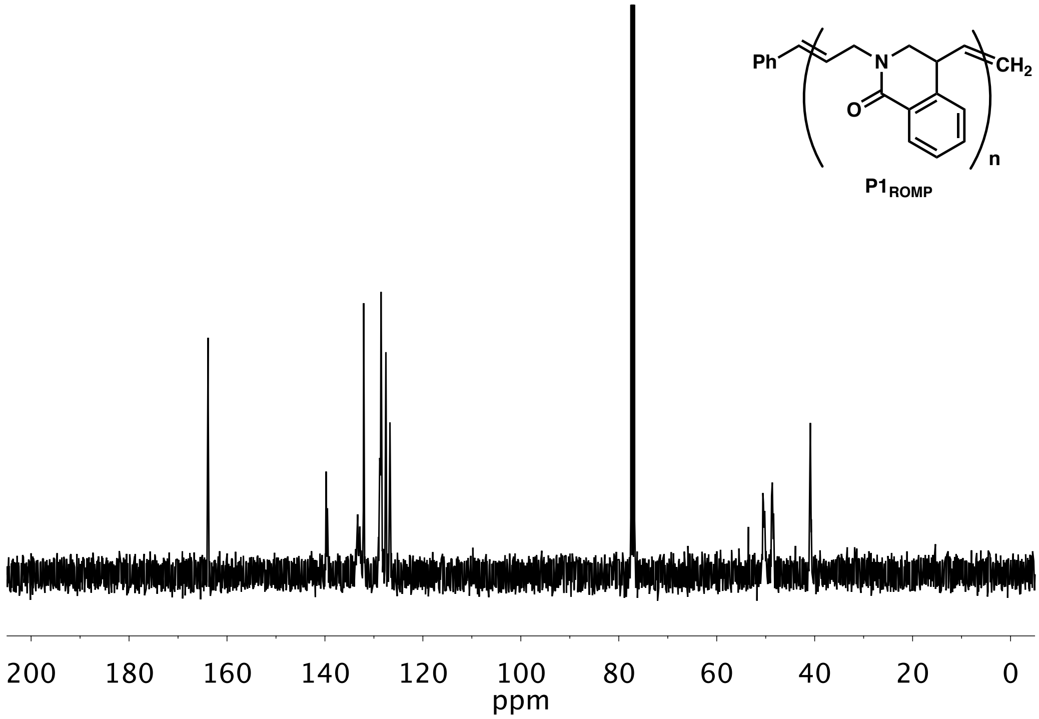

Figure S2. ^{13}C NMR (101 MHz, $CDCl_3$, 25 °C) of polymer **P1$_{ROMP-100}$**.

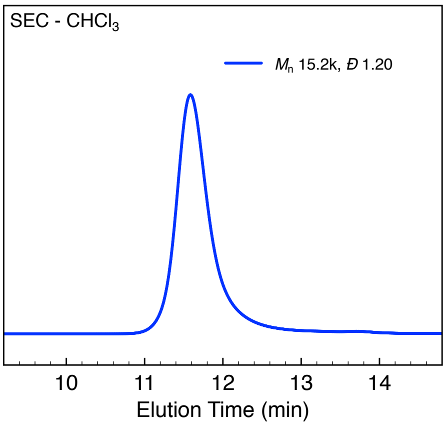

Figure S3. SEC trace of polymer **P1**$_{\mathbf{ROMP-100}}$.

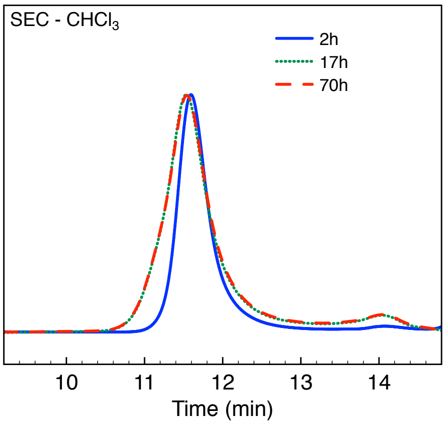

Figure S4. SEC traces of ROMP of **1** (targeting DP 100) at extended reaction times.

Halide-Rebound Polymerization (HaRP) of 1

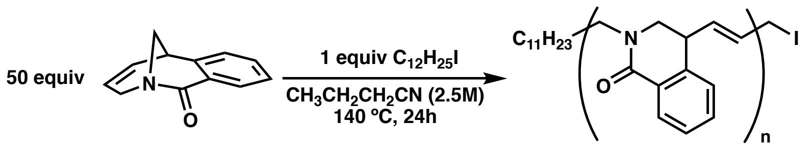

1 **P1**$_{\mathbf{HaRP}}$

Halide-rebound polymerization of **1** was performed according to our reported method.[5] **1** (138.9 mg, 0.75 mmol, 50 equiv), 25 μL of 0.6 mol/L stock 1-iodododecane solution in butyronitrile (0.0015 mmol 1-iodododecane, 1 equiv) and butyronitrile were added to an oven-dried microwave vial, forming the reaction solution with 2.5 M of **1**. The vial was sealed, covered by aluminum foil, and placed into a pre-heated oil bath (140 °C) for 24 hours with stirring. The reaction was then cooled to room temperature, and the solvent was removed under vacuum. A sample of the crude product was taken for ^{1}H NMR to determine the conversion of **1**, and the molecular weight (M_n) and dispersity ($Đ$) were determined by SEC analysis (74% conversion, M_n: 4.7 kg/mol, $Đ$: 1.94). The crude polymer was then precipitated by slowly adding a concentrated DCM solution into a Et_2O/hexane mixture with vigorous stirring.

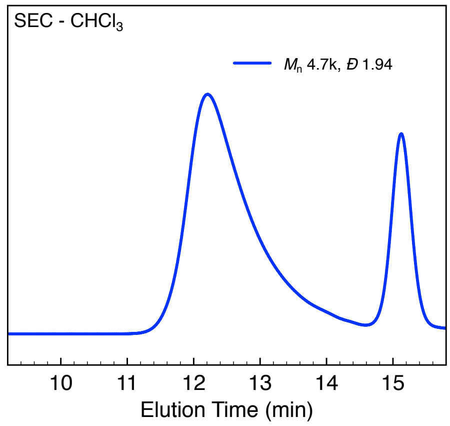

Figure S5. SEC trace of HaRP of **1** targeting DP 50.

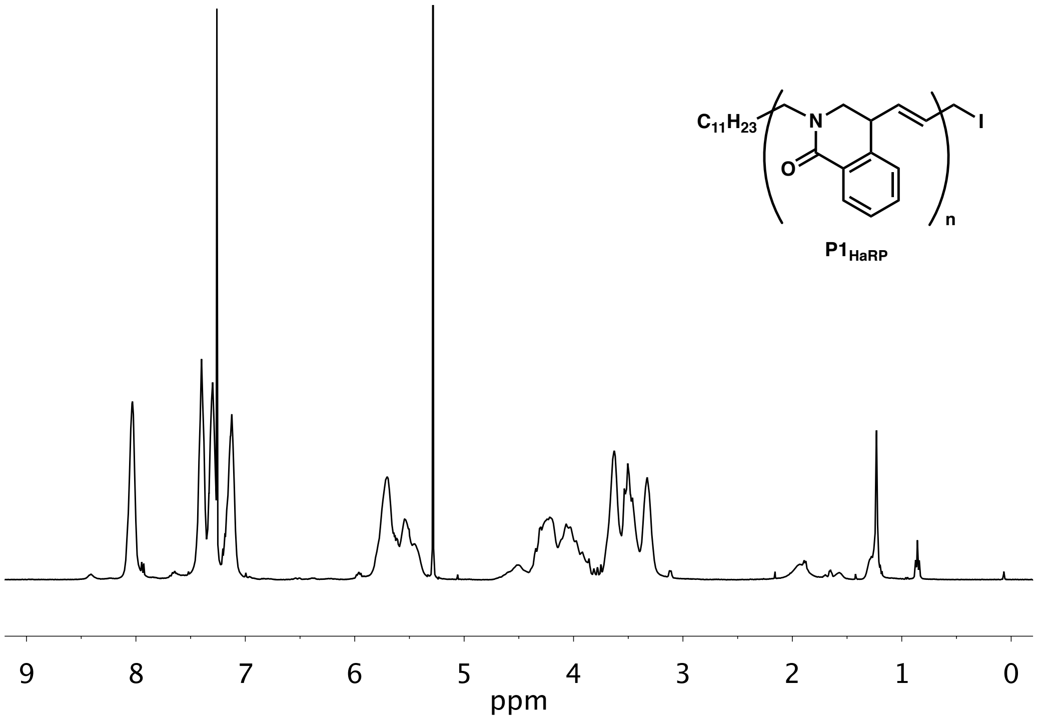

Figure S6. ^{1}H NMR (400 MHz, $CDCl_3$, 25 °C) of **P1$_{HaRP}$**.

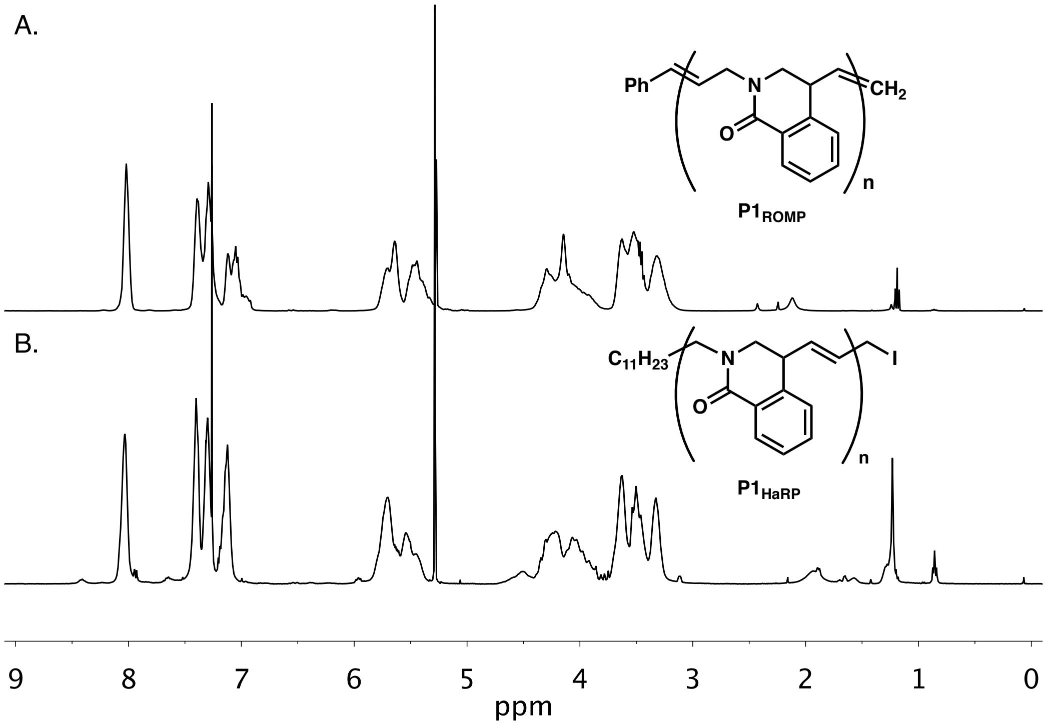

Figure S7. Stacked ^{1}H NMR of (A) **P1**$_{\mathbf{ROMP}}$ and (B) **P1**$_{\mathbf{HaRP}}$.

Attempted Polymerization of Ketone 2

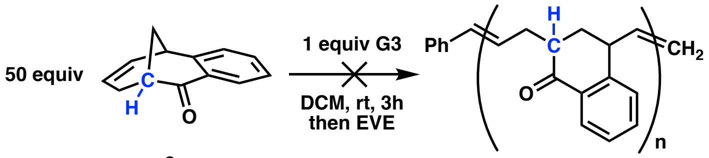

Ketone **2** (18.4 mg, 0.1 mmol, 50 equiv) was dissolved in DCM (450 μL) in a 4 mL vial. A solution of **G3** (1.45 mg, 0.002 mmol, 1 equiv) in DCM (50 μL) was rapidly added to the ketone solution with stirring. After stirred at room temperature for 3 h, the reaction was quenched by EVE (100 μL) and the solvent was removed under vacuum. The crude mixture was analyzed by ^{1}H NMR, and **2** remained intact with no polymerization occurring.

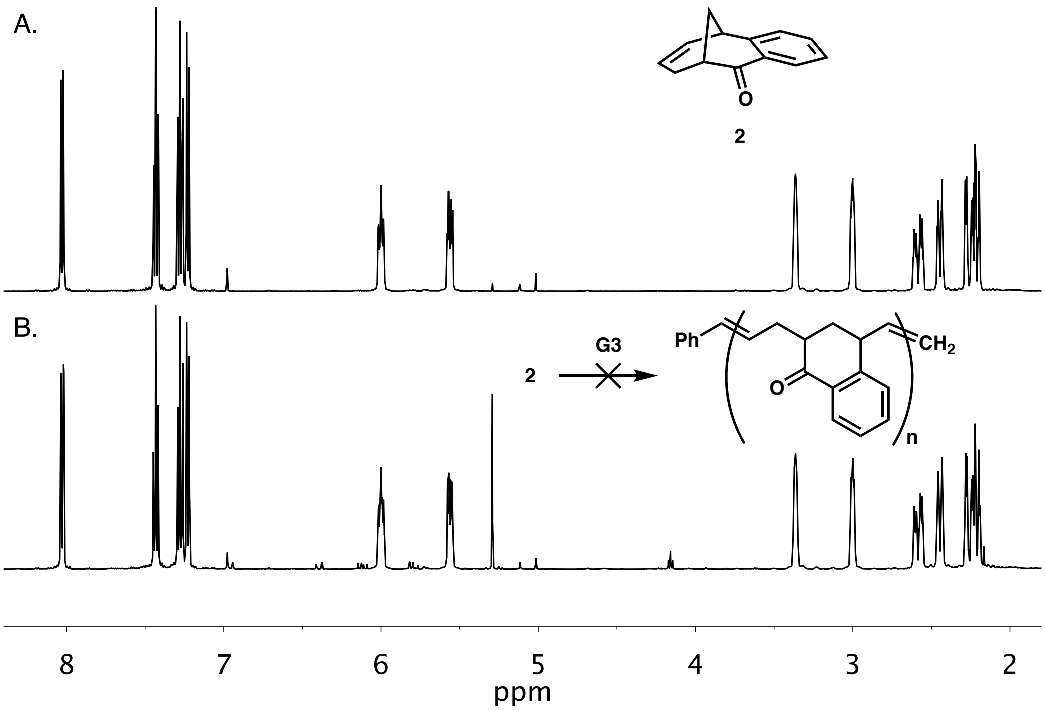

Figure S8. (A) ^{1}H NMR (500 MHz, $CDCl_3$, 25 °C) of **2**. (B) ^{1}H NMR (500 MHz, $CDCl_3$, 25 °C) of attempted polymerization of **2**.

Polymerization Kinetic Studies

Twisted amide **1** (27.78 mg, 0.15 mmol, 100 equiv) was dissolved in DCM (700 µL) in a 4 mL vial. A solution of **G3** (1.09 mg, 0.0015 mmol, 1 equiv) in DCM (50 µL) was rapidly added to the twisted amide solution with stirring ([**1**] = 0.2 M, [**G3**] = 0.002 M). When the reaction solution was stirred at room temperature, aliquots were taken at 5 min, 10 min, 20 min, 40 min and 60 min, and were analyzed by SEC and ^{1}H NMR analyses, respectively. $[M]_0/[M]$ was calculated by the relative integration of monomer olefin proton (5.99 ppm) and the protons *ortho*- to carbonyl in both polymer and monomer (8.20-7.90 ppm) on ^{1}H NMR spectra of aliquots taken at different time points.

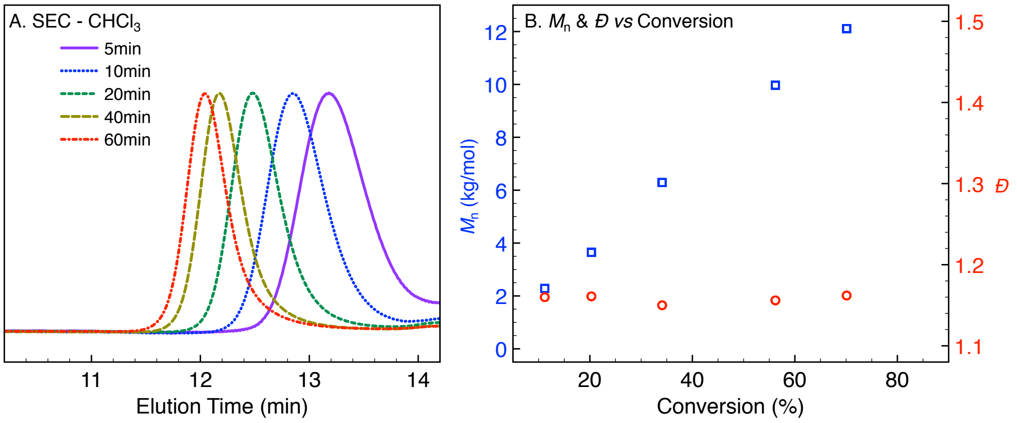

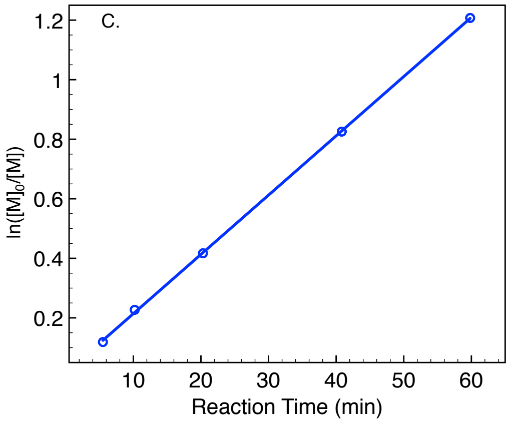

Figure S9. (A) Size-exclusion chromatograms of samples taken from different time points. (B) M_n-conversion correlation (blue) and *Đ*-conversion correlation (red). (C) First-order kinetic plot, k_{obs} = 0.0199 min^{-1}, k = k_{obs}/[**G3**] = 0.166 M^{-1}·s^{-1}.

Kinetic Studies at Variable Temperatures

Under nitrogen, twisted amide **1** (18.52 mg, 0.1 mmol, 100 equiv) was dissolved in degassed $CDCl_3$ (450 μL) in an NMR tube, and the solution was cooled to 0 ºC. A pre-cooled (0 ºC) solution of **G3** (0.73 mg, 0.001 mmol, 1 equiv) in degassed $CDCl_3$ (50 μL) was rapidly added to the NMR tube. The NMR sample was kept at 0 ºC before measured in the NMR instrument at indicated temperature (from 15 ºC to 40 ºC with 5 ºC increments). At different time points (10 sec, 5, 10, 15, 20, 25, 30, 40, 50, 60, 80, 100, 120 min), quantitative ^{1}H NMR data were collected. $[M]_0/[M]$ was calculated by the relative integration of monomer olefin proton (5.99 ppm) and the protons *ortho-* to carbonyl in both polymer and monomer (8.20-7.90 ppm), and k_{obs} was calculated from the linear regression slope of $\ln([M]_0/[M])$ vs. time plot.

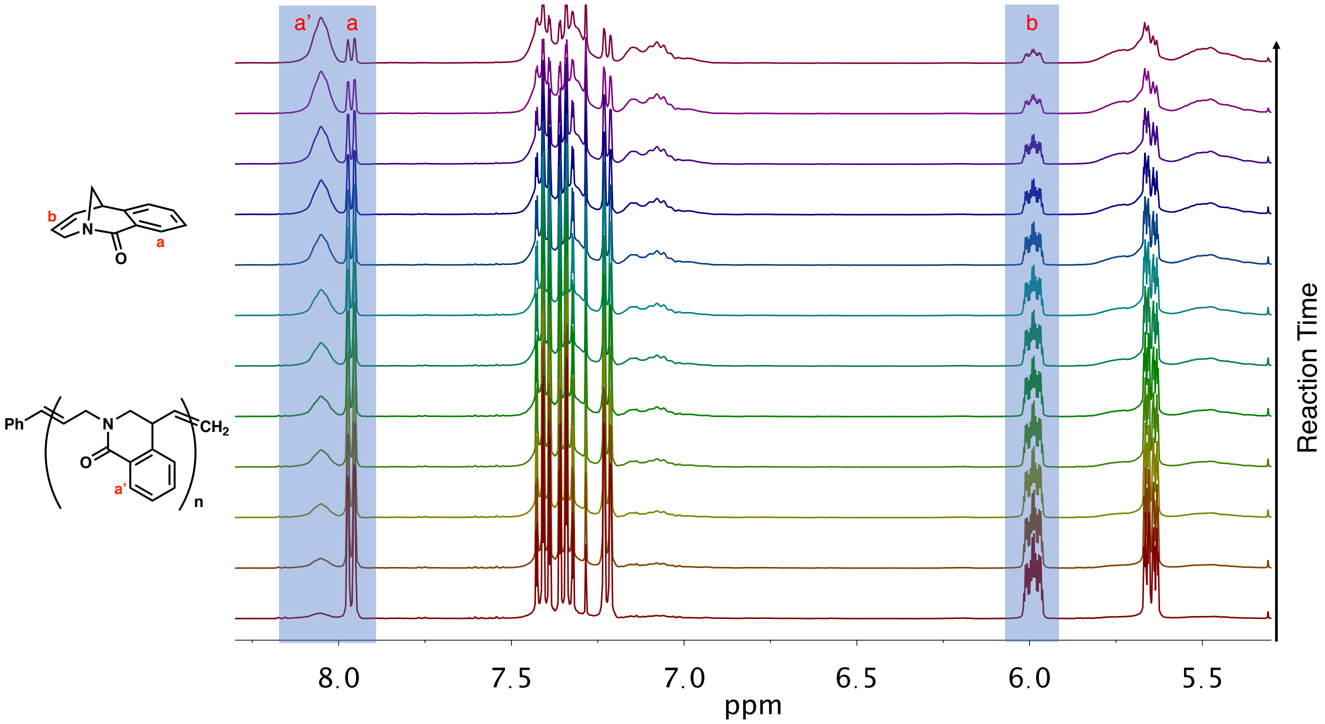

Figure S10. Stacked ^{1}H NMR for the kinetics of ROMP of **1** at 25 ºC (targeting DP 100) at different time points (10 sec, 5, 10, 15, 20, 25, 30, 40, 50, 60, 80, 100 min). For kinetic ^{1}H NMR spectra at other temperatures, please see NMR files in accompanying MNova file.

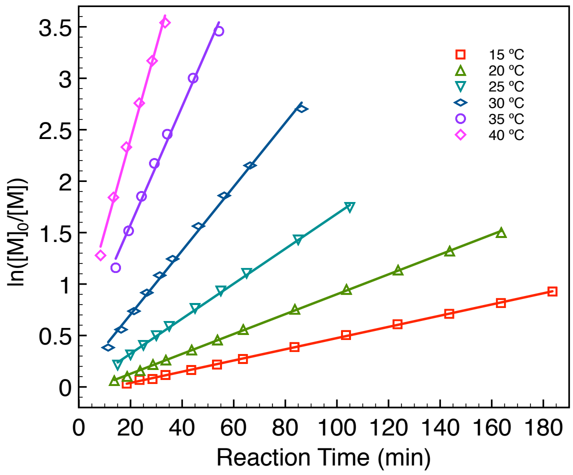

Figure S11. Plots of ln([M]$_0$/[M]) vs. time at different temperatures.

Table S2 Rate constants of polymerization of **1** at different temperatures[a]

Entry	Temperature	k_{obs} (min^{-1})	k_{obs} (s^{-1})	R^2 value
1	15 ºC	0.00544	0.0000907	0.9997
2	20 ºC	0.00966	0.000161	0.9998
3	25 ºC	0.0172	0.000283	0.9987
4	30 ºC	0.0310	0.000517	0.9976
5	35 ºC	0.0613	0.00102	0.9950
6	40 ºC	0.0898	0.00150	0.9944

[a] Polymerizations were carried out with [**1**] = 0.2 M and [**G3**] = 0.002 M in $CDCl_3$.

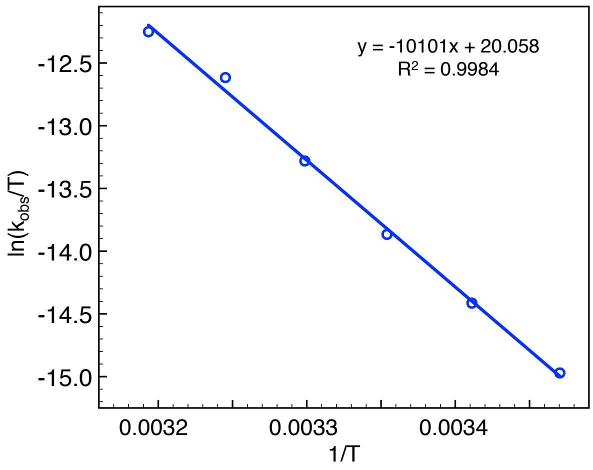

Figure S12. Eyring plot of ROMP of **1**. Values of k_{obs} (s^{-1}) and T (K) were used. $\Delta H^‡$ = 84.0 kJ·mol^{-1}, $\Delta S^‡$ = -30.8 J·mol^{-1}·K^{-1}, $\Delta G^‡$ = 93.0 kJ·mol^{-1} (at 20 °C)

Regioregularity Studies

Synthesis of Polymer P2 through halide-rebound polymerization (HaRP)

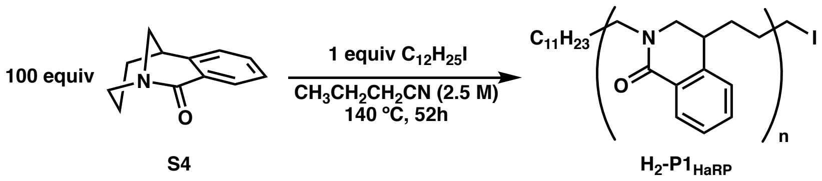

According to our reported method, twisted amide monomer **S4** was synthesized and corresponding HaRP was performed to synthesize polymer **H_2-P1$_{HaRP}$** (targeting DP 100).[5] The NMR data of **S4** and **H_2-P1$_{HaRP}$** matched those from literature.

Reduction of Polymer P1$_{ROMP}$ to Synthesize H_2-P1$_{ROMP}$

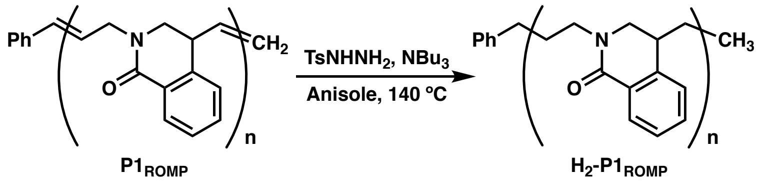

Reduction of polymer **P1$_{ROMP}$** (an example: M_n 4.6 kg/mol, $Đ$ 1.20) with diimide was performed according to a literature method[6]. In a 8 mL vial, polymer **P1$_{ROMP}$** (37.1 mg, 0.2 mmol of olefin, 1 equiv), *p*-toluenesulfonylhydrazide (137.8 mg, 0.74 mmol, 3.7 equiv), tributylamine (140.9 mg, 0.76 mmol, 3.8 equiv) and a small amount of BHT (*ca.* 3 mg) were dissolved in anisole (2 mL). The reaction mixture was connected to vacuum and refilled with nitrogen (repeated for 3 times), before heated up to 140 °C and stirred overnight. The reaction was then cooled down to room temperature, and diluted with DCM, and washed with 0.1M HCl, aqueous sat. $NaHCO_3$, water and brine. The organic phase was dried (Na_2SO_4), filtered and concentrated. The crude mixture was precipitated for three times by slowly adding a concentrated DCM solution into an Et_2O/hexane mixture with vigorous stirring, and then dried under vacuum. The purified reduced polymer **H_2-P1$_{ROMP}$** was then characterized using NMR, SEC and MALDI-TOF-MS analyses (M_n: 4.5 kg/mol, $Đ$: 1.20).

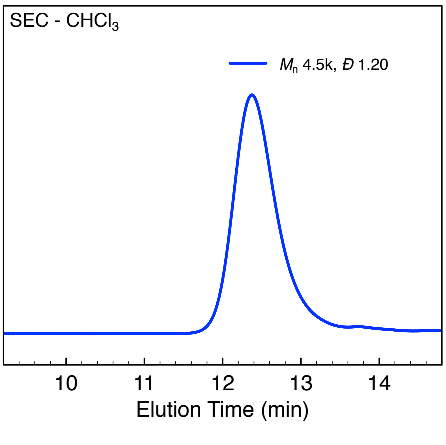

Figure S13. SEC trace of **H_2-P1$_{ROMP}$**.

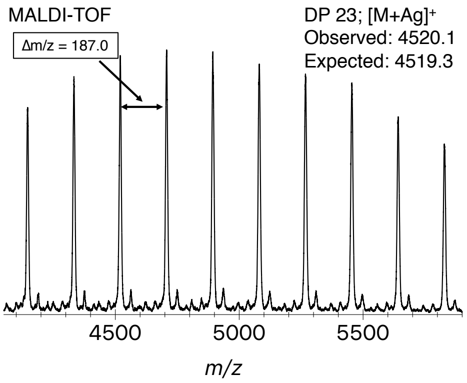

Figure S14. MALDI-TOF MS data of **H_2-P1$_{ROMP}$** (M_n: 4.5 kg/mol, *Đ*: 1.20).

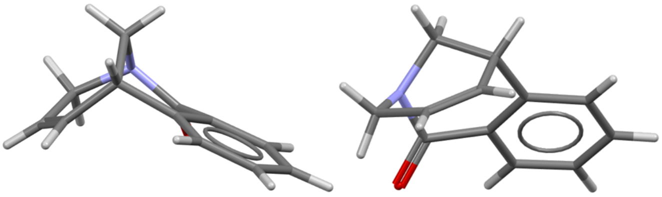

Figure S15. Crystal structure of twisted amide **1** showing limited steric bias on either side of the C=C double bond (taken from Ref. [2b]).

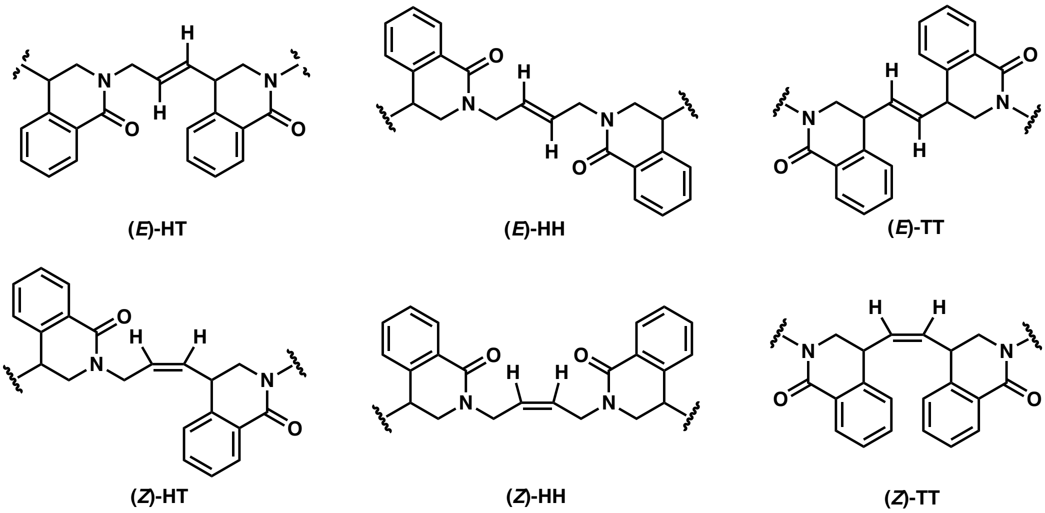

Figure S16. Possible head-to-tail (HT), head-to-head (HH) and tail-to-tail connectivities in **P1$_{ROMP}$**.

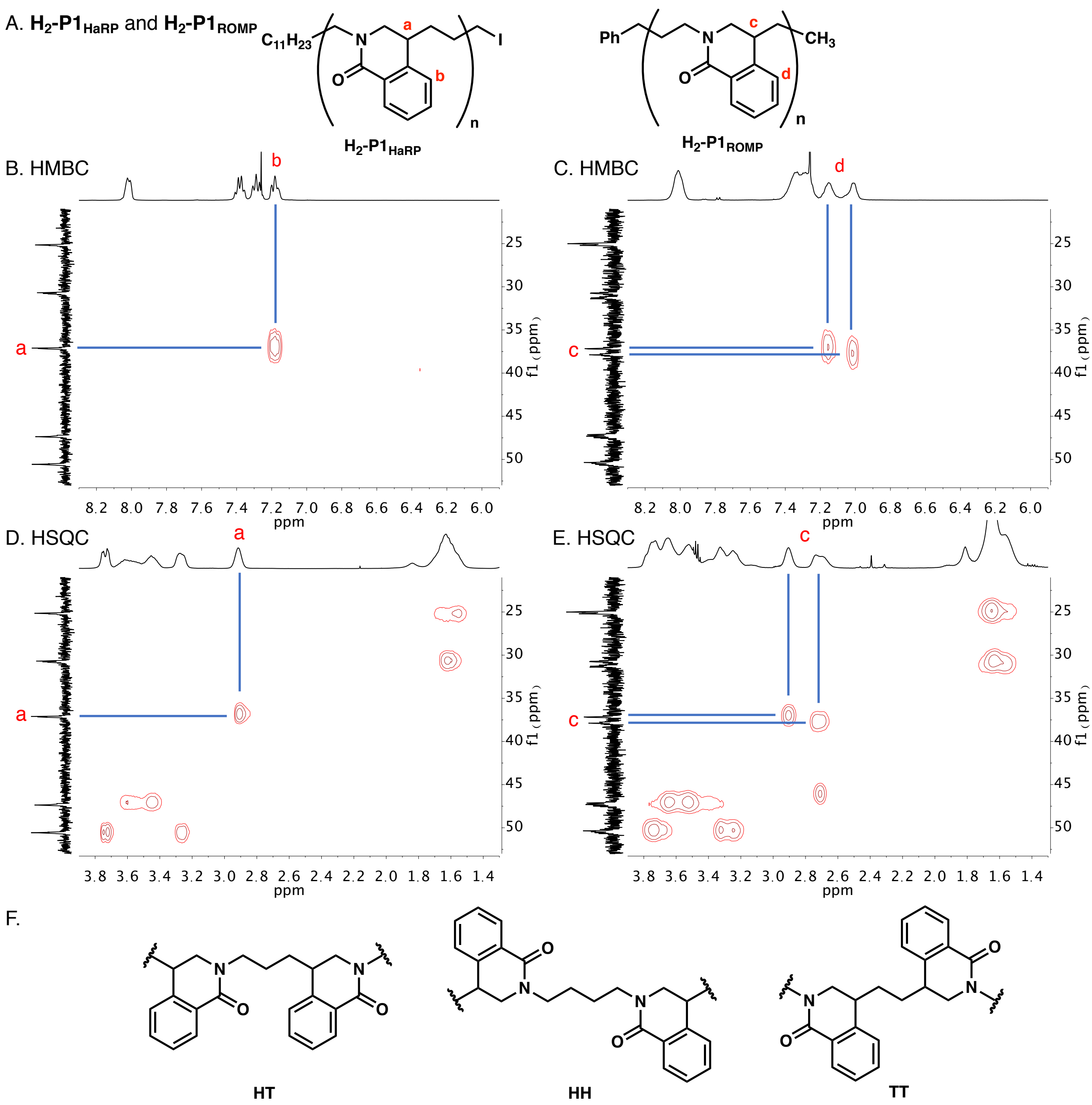

Figure S17. (A) Structures of **H_2-P1$_{HaRP}$** and **H_2-P1$_{ROMP}$**. HMBC of (B) **H_2-P1$_{HaRP}$** and (C) **H_2-P1$_{ROMP}$**. HSQC of (D) **H_2-P1$_{HaRP}$** and (E) **H_2-P1$_{ROMP}$**. (F) Possible head-to-tail (HT), head-to-head (HH) and tail-to-tail (TT) connectivities in **H_2-P1$_{ROMP}$**.

The assignments of aromatic proton **b** and proton **d** were determined by COSY experiments of **H_2-P1$_{HaRP}$** and **H_2-P1$_{ROMP}$** (Figure S38 and S43), respectively. In addition, based on HMBC spectra of **H_2-P1$_{HaRP}$** and **H_2-P1$_{ROMP}$**, (Figure S17B and C), carbon **a** (37.1 ppm) correlating with aromatic proton **b** is assigned as the benzylic carbon in **H_2-P1$_{HaRP}$**, and carbon **c** (37.2, 37.9 ppm) correlating with aromatic proton **d** is assigned as the benzylic carbon in **H_2-P1$_{ROMP}$**. Therefore, HSQC spectra of **H_2-P1$_{HaRP}$** and **H_2-P1$_{ROMP}$** (Figure S17D and E) confirm the assignments of proton **a** (2.91 ppm) and proton **c** (2.91, 2.73 ppm) as the benzylic protons in **H_2-P1$_{HaRP}$** and **H_2-P1$_{ROMP}$**, respectively. The existence of two types of benzylic protons **c** in **H_2-P1$_{ROMP}$** is consistent with the two possible types of connection (tail-to-tail and tail-to-head) on the benzylic position (Figure S17F).

Polymer Thermal Properties

TGA curves of purified **P1$_{ROMP}$** and **H$_2$-P1$_{ROMP}$** samples were obtained in a nitrogen atmosphere at a heating rate of 10 °C/min.

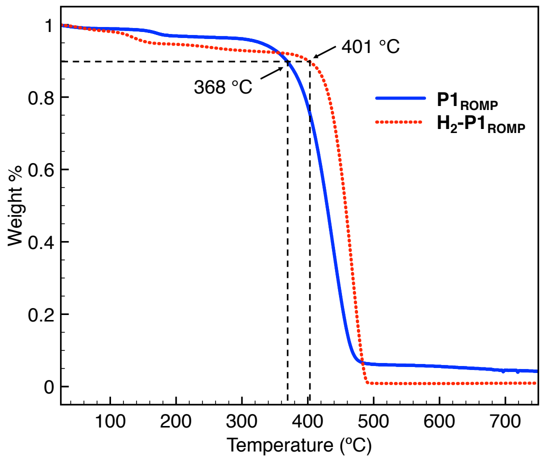

Figure S18. TGA of **P1$_{ROMP}$** (M_n 15.2 kg/mol, $Đ$ 1.20) and **H$_2$-P1$_{ROMP}$** (M_n 15.4 kg/mol, $Đ$ 1.27).

DSC analysis of purified **P1$_{ROMP}$** and **H$_2$-P1$_{ROMP}$** samples were performed under nitrogen flow (-20 °C to 220 °C, heating rate: 10 °C/min, cooling rate: 10 °C/min). A glass transition temperature (T_g) was found at 158 °C.

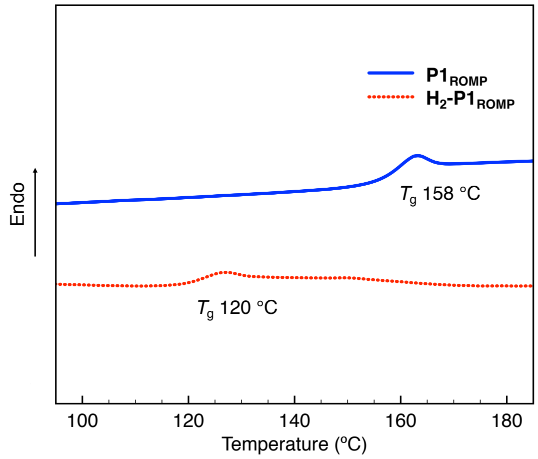

Figure S19. DSC of **P1$_{ROMP}$** (M_n 59.6 kg/mol, $Đ$ 1.58) and **H$_2$-P1$_{ROMP}$** (M_n 14.4 kg/mol, $Đ$ 1.23).

Computational Studies

Computational Methods. All the calculations were carried out using Psi4 with the standard grid size (75,302).[7] All of the geometry optimizations and frequency analysis were performed at the B3LYP-D3MBJ/6-311++G(d,p) level of theory in the gas phase. The absence of imaginary frequencies was used to characterize the structures as minima on the potential energy surface. All of the optimized geometries were verified as minima (no imaginary frequencies). Electronic and thermal energies were calculated for all structures. Energetic parameters were calculated under standard conditions (298.15 K and 1 atm).[8] Strain energies and resonance energies were calculated using isodesmic reactions based on total energies (with zero-point energy and thermal corrections) for optimized structures. Gibbs free energies were determined using vibrational frequency calculations.

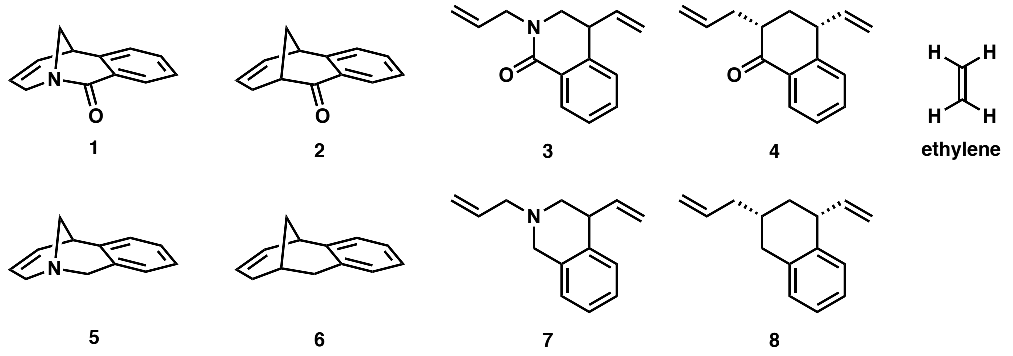

Figure S20. Structures calculated in this study.

Table S3. Total energies (without and with zero-point energy (ZPE) and thermal corrections) and Gibbs free energies calculated for optimized structures using B3LYP-D3MBJ/6-311++G(d,p).

Compound	total energy (au)	total energy (ZPE correction) (au)	total energy (ZPE and thermal corrections) (au)	Gibbs free energy (au)	total energy (kcal/mol)	total energy (ZPE and thermal corrections) (kcal/mol)	Gibbs free energy (kcal/mol)
**1**	-594.086519	-593.881864	-593.874841	-593.886456	-372795.232	-372662.402	-372669.690
**2**	-578.050137	-577.834186	-577.826850	-577.839417	-362732.242	-362592.126	-362600.013
**3**	-672.732575	-672.474993	-672.463448	-672.485681	-422146.418	-421977.538	-421991.489
**4**	-656.677080	-656.408693	-656.396918	-656.419911	-412071.435	-411895.630	-411910.058
**5**	-520.028001	-519.805486	-519.798844	-519.809882	-326322.771	-326178.973	-326185.899
**6**	-504.005878	-503.771588	-503.764737	-503.777002	-316268.729	-316117.410	-316125.106
**7**	-598.658221	-598.382918	-598.371918	-598.393036	-375664.020	-375484.362	-375497.614
**8**	-582.635347	-582.348414	-582.337186	-582.359324	-365609.506	-365422.408	-365436.300
**Ethylene**	-78.620522	-78.569728	-78.569519	-78.569773	-49335.164	-49303.159	-49303.318

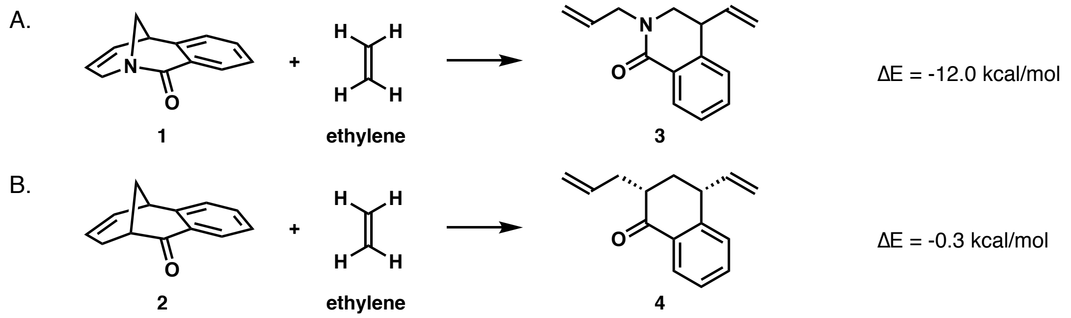

Figure S21. Ring strain energies obtained from isodesmic ring-opening metathesis reactions. (A) **1** + ethylene, (B) **2** + ethylene.

A.

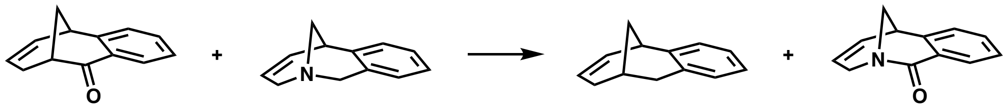

2 5 6 1

-RE_1 = ΔE = -8.7 kcal/mol

B.

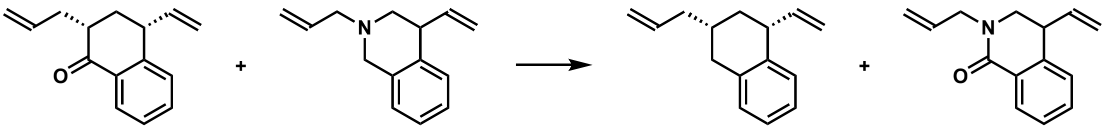

4 7 8 3

-RE_{S5} = ΔE = -20.0 kcal/mol

Recovery of resonance energy from **1** to **3** after ring-opening = RE_{S5} - RE_1 = 11.3 kcal/mol

Figure S22. Resonance energies of (A) twisted amide **1** and (B) amide **3** determined by COSNAR method.[8c,8d]

1

Energy: -594.086519 au

Energy including zero-point energy and thermal corrections: -593.874841 au

Geometry:

C	0.900708115883	-1.993574027020	-0.884705617429
C	0.292871915262	-0.920262100065	-0.235471620759
C	1.074773533704	0.085910507887	0.359350389761
C	2.461390177780	0.000523573899	0.286521000468
C	2.288093556447	-2.081260112195	-0.934636160682
C	3.066502846961	-1.080209593094	-0.354811176161
H	4.147815252217	-1.141975773252	-0.398531785323
H	2.762260843698	-2.918684567525	-1.432245735660
C	0.342802502963	1.199404380516	1.090869508133
C	-0.157937665460	2.296753690995	0.163666674167
C	-1.422043394574	2.357834828343	-0.263201738100
H	0.558615366233	3.050650855984	-0.145703445775
C	-2.442287283656	1.285545962354	0.057104991221
N	-1.761613093933	0.119619678879	0.636933416733
C	-1.188010880038	-0.719014823166	-0.329836417661
C	-0.872725309797	0.534073123874	1.730507061998
H	-0.588012293903	-0.336172374595	2.323290761968
H	-1.426570313973	1.221022420347	2.372454401765
H	1.007149907126	1.633593313656	1.843790866946
O	-1.836261896493	-1.165703000060	-1.258598856336
H	-3.202117792192	1.652237780142	0.755856486523
H	-2.961556220243	0.962710414192	-0.845378372733
H	-1.747278986663	3.181093500494	-0.892341539039
H	0.272555877678	-2.738526186911	-1.357343512273
H	3.071508244013	0.773838400034	0.740825940986

2

Energy: -578.050137 au

Energy including zero-point energy correction: -577.826850 au

Geometry:

C	0.568380598452	-0.600488190367	0.493799465529

C	0.915077671601	0.609435029124	-0.131804756153
C	2.247025219896	0.838947141926	-0.471014597377
C	3.223295437909	-0.114937229342	-0.193713318080
C	1.553602439895	-1.554337453433	0.770343463823
C	2.877541458109	-1.315734360964	0.427921517915
C	-0.177200597314	1.609975935698	-0.456237357906
C	-0.837656688414	1.222153036884	-1.765285684971
C	-1.957536244508	0.502321728322	-1.814853390406
C	-2.683187754853	0.014131976968	-0.590624638765
C	-1.876231941559	0.232014638650	0.707606074240
C	-0.845299093248	-0.885890364061	0.872381554391
C	-1.210895554138	1.610981983543	0.676676056266
H	-0.721579472128	1.828423523273	1.629842875772
H	-1.962763694227	2.385479153142	0.501478860217
O	-1.184793209962	-1.974970073354	1.298284351435
H	0.274785548944	2.600077652025	-0.560916142064
H	1.253140622909	-2.475633946861	1.253872059305
H	4.256094273735	0.079414707837	-0.459804821007
H	3.638588718708	-2.056326685268	0.642811933943
H	2.521456747296	1.771610752588	-0.952171055842
H	-2.553473362394	0.133079051784	1.558578773551
H	-2.933540522466	-1.045881801189	-0.695419179565
H	-3.641803377708	0.541414408054	-0.504530721271
H	-0.335920759599	1.520092702424	-2.680417965349
H	-2.387015187895	0.241621152809	-2.777781658419

3

Energy: -672.732575 au

Energy including zero-point energy correction: -672.463448 au

Geometry:

C	0.471261754683	-2.294818767968	-1.341537621280
C	0.071390465899	-1.107131515263	-0.725081288027
C	0.215350830778	0.117929901746	-1.392634481206
C	0.754641826089	0.129336011013	-2.678703281515
C	1.158353474829	-1.056492705011	-3.287197189483
C	1.018817020063	-2.271600818188	-2.618041684050
H	0.335808037944	-3.221767165890	-0.799197336370
H	0.857922096491	1.071470617810	-3.203334117829
H	1.332114535119	-3.194265247553	-3.091846648861
H	1.581780600311	-1.031137021517	-4.284768572602
C	-0.538735726759	-1.196433257673	0.638013110360
N	-0.749721818672	-0.001842281107	1.276944269619
C	-0.111147049585	1.223800979009	0.806322368533
C	-0.265389675435	1.384894051386	-0.708598293887
H	0.953444347928	1.235618619112	1.075826707411
H	-0.582526765213	2.065640157857	1.315535803926
C	0.456891293216	2.619494410913	-1.168107902132
H	-1.334972080000	1.501647100351	-0.925089595637
C	-0.128353781919	3.671048382727	-1.732951693074
H	1.532889527502	2.624637911272	-1.004728405501

H	0.438947430943	4.544974822356	-2.031538261240
H	-1.197823992575	3.691907501138	-1.918591458650
C	-1.228391124137	-0.023541738594	2.660420824780
C	-0.095739157087	-0.026111715695	3.649080314692
H	-1.828345024441	-0.927108874271	2.771306467390
H	-1.869888986275	0.848281474628	2.816039288002
C	0.120984189494	0.935913995016	4.540947710756
H	0.570391806924	-0.883199111250	3.590629452228
H	-0.534245553282	1.799374296115	4.612022395777
H	0.949569556703	0.892208723307	5.238341889719
O	-0.844974637763	-2.272856845839	1.139269580139

4

Energy: -656.677080 au

Energy including zero-point energy correction: -656.396918 au

Geometry:

C	-0.882660972904	-1.933445303973	-1.851687466874
C	-0.446564971899	-0.961463315958	-0.941032301468
C	0.124332494123	0.236268136461	-1.406794768466
C	0.231321190659	0.433151962425	-2.786426506768
C	-0.211224126660	-0.532618973862	-3.683528283221
C	-0.768766502823	-1.724265038393	-3.216753453613
H	-1.312590985556	-2.844364852783	-1.454467055435
H	0.672344999537	1.351272704379	-3.155105831045
H	-1.111231930778	-2.478076500926	-3.915641749813
H	-0.117367212464	-0.358144436240	-4.749381160499
C	-0.625146399769	-1.235482914858	0.515018534090
C	-0.313968551530	-0.089470774518	1.469920361573
C	0.884693678055	0.708176178062	0.947814999591
C	0.603995089643	1.307207415298	-0.438047026313
H	1.761378901156	0.051226212420	0.891500580276
H	1.134541253149	1.516220348963	1.637503374473
C	1.816075803940	2.037059530703	-0.948385583063
H	-0.208715810741	2.037811422156	-0.324032454808
C	1.882261807761	3.348902600073	-1.154161242570
H	2.690960307003	1.416845188825	-1.134896502576
C	-0.150029428004	-0.589021741552	2.914014226669
C	-0.087133554700	0.523668078247	3.919011771947
H	0.763740531320	-1.193690992724	2.967034476349
H	-0.978834697566	-1.258834192611	3.146790531920
C	-1.019185363557	0.758113623527	4.839731978566
H	0.781794860152	1.177101558600	3.878238533660
O	-1.022252365941	-2.315075030251	0.912339662992
H	-1.195188204061	0.569336031423	1.433663006510
H	-1.900121960983	0.128868786911	4.924780124498
H	-0.933294629048	1.579892924977	5.541570493940
H	2.792885349406	3.825994390369	-1.497566034106
H	1.024295642825	3.992170855761	-0.983519097597

5

Energy: -520.028001 au

Energy including zero-point energy correction: -519.798844 au

Geometry:

C	0.745310047825	-2.172221805214	-0.703832811386
C	0.125663986954	-1.071256967701	-0.110777160420
C	0.894250554860	0.050328265171	0.220967233897
C	2.263628208363	0.051538568406	-0.049485540273
C	2.108405674099	-2.161932703418	-0.984294053875
C	2.873253135304	-1.045050103301	-0.651385194170
H	2.854329809851	0.922852031330	0.215350395929
H	3.937069848050	-1.030207947787	-0.858073027871
H	2.572175159970	-3.020343246088	-1.456353080383
C	0.200505527048	1.258759395091	0.822778067166
C	-0.293102475700	2.159604324268	-0.292095724561
C	-1.522500770004	2.027267907333	-0.784880591620
H	0.396491232809	2.891023760060	-0.700900466260
C	-2.498010361832	1.007913064772	-0.249871364698
N	-1.953269883738	0.095766386112	0.759188004836
C	-1.354868285129	-1.134372166078	0.242853581578
C	-1.014909921684	0.789359601663	1.639837117708
H	-0.707000747195	0.107564827400	2.437438370104
H	-1.520120699706	1.643211296055	2.098382589765
H	0.907696083417	1.804444615394	1.455146567177
H	-3.353506181092	1.540782012578	0.190138172878
H	-2.909287551209	0.412754579307	-1.070322747273
H	-1.877907603399	2.682180512967	-1.575569613258
H	0.151904734076	-3.048071863382	-0.949688657123
H	-1.930617119118	-1.466548727556	-0.624199380473
H	-1.477812696475	-1.911869085167	1.008592550554

6

Energy: -504.005878 au

Energy including zero-point energy correction: -503.764737 au

Geometry:

C	0.479522954469	-0.972288124413	-0.151184003722
C	0.777529345457	0.281565428065	0.395844278782
C	2.100168560441	0.728044042756	0.425308879422
C	3.131125806405	-0.051549367001	-0.088663108285
C	1.523331239285	-1.750877241747	-0.659125422802
C	2.838729819113	-1.298837683519	-0.637653484759
C	-0.356428895845	1.157294146775	0.895940804289
C	-0.898887386236	1.956218591461	-0.271476521782
C	-1.945692807088	1.546078008177	-0.984638532734
C	-2.703787769129	0.282242951937	-0.683044426952
C	-2.039702087853	-0.600769576040	0.391163072038
C	-0.933314196660	-1.527349339264	-0.159415576361
C	-1.466525891893	0.288632694094	1.500962724348
H	-2.818864827068	-1.244147363970	0.810086688908
H	-1.061488164284	-0.322182188864	2.313315465015

H	-2.251299951083	0.925305964301	1.919680992978
H	-3.716402552048	0.552388133808	-0.353265831838
H	-2.842531101805	-0.293859639260	-1.605183362473
H	-1.189722530357	-1.843764541091	-1.175798875119
H	-0.923276896933	-2.444019831705	0.441990426580
H	0.037410364398	1.844837522476	1.650091334585
H	1.298381339486	-2.727476009936	-1.077148817342
H	3.631494239024	-1.917427537418	-1.042286420307
H	4.153088802575	0.308263844857	-0.059735644955
H	2.319157889278	1.699886719987	0.856402680032
H	-0.367513360309	2.862299300480	-0.545592478032
H	-2.294106449493	2.142619451768	-1.823201049515

7

Energy: -598.658221 au

Energy including zero-point energy correction: -598.371918 au

Geometry:

C	0.323437076624	-2.417898645746	-1.400850300436
C	0.045994546132	-1.263819583852	-0.663539411614
C	0.032255709232	-0.020858517620	-1.306592088689
C	0.291292488590	0.035657785057	-2.680151026654
C	0.563722456594	-1.116188412093	-3.408752445542
C	0.582124249491	-2.352427563849	-2.764585175828
H	0.331220808751	-3.379040807363	-0.896080936837
H	0.280325153063	0.999217269524	-3.176369080652
H	0.791571213972	-3.257898359516	-3.322165131616
H	0.760962338832	-1.051155763998	-4.472523471753
C	-0.208192799334	-1.380029356768	0.821165857589
N	-0.868026538486	-0.197943541504	1.352358718166
C	-0.154367845408	1.014084688745	0.977493588635
C	-0.287979736198	1.251044107217	-0.532863269535
H	0.913884158187	0.968277731138	1.254276965989
H	-0.585724913310	1.860960024245	1.514993315051
C	0.578003219980	2.405260622796	-0.955777244983
H	-1.334782144369	1.509292776317	-0.729814234609
C	0.122560731985	3.561931246477	-1.428567611003
H	1.650342722062	2.254050740219	-0.846269256671
H	0.792310727616	4.368911325460	-1.703096627049
H	-0.940857682158	3.739244916706	-1.558334665054
C	-1.174567062251	-0.286044463121	2.781987397636
C	0.016277277936	-0.271045634673	3.708782716800
H	-1.747408093359	-1.208235104622	2.932069765275
H	-1.836521182621	0.548105570309	3.034201407583
C	0.248861530875	0.683410180640	4.605355372185
H	0.721119054977	-1.093869570107	3.616864026468
H	-0.431855800727	1.521497339090	4.725866460156
H	1.117641989629	0.662862185458	5.253761852285
H	0.760354515439	-1.574060236430	1.320357054366
H	-0.843726206942	-2.249401350146	1.015494496734

8

Energy: -582.635347 au

Energy including zero-point energy correction: -582.337186 au

Geometry:

C	-0.900695314782	-2.031537339950	-1.876699199270
C	-0.431160804200	-1.133206336674	-0.911914804545
C	0.085729287389	0.103147169371	-1.325648252645
C	0.114048254919	0.405100714619	-2.692290704616
C	-0.353174084168	-0.495092745993	-3.641950423863
C	-0.863509141509	-1.725324413235	-3.231170905183
H	-1.303499936038	-2.985797860950	-1.551905602274
H	0.513141861820	1.362056089448	-3.008090127497
H	-1.233306356128	-2.437059743959	-3.960238414072
H	-0.320386393058	-0.239300809831	-4.694714474219
C	-0.482456643718	-1.524509572500	0.548182523431
C	-0.390025866140	-0.333474192390	1.502846953944
C	0.790069538621	0.540308543239	1.078010050689
C	0.575460048203	1.142292419526	-0.320746221261
H	1.701764689887	-0.071544358826	1.077838169270
H	0.957507327526	1.358007583379	1.782072668622
C	1.833065684810	1.823270592774	-0.787646831516
H	-0.206615351725	1.907903561229	-0.233174349227
C	1.968818712569	3.133648885776	-0.969751888478
H	2.680619039169	1.163937273983	-0.967130742296
C	-0.284643093767	-0.817012314337	2.961026298936
C	-0.374791571562	0.283045179386	3.977230037913
H	0.669747013631	-1.348269756482	3.077664867361
H	-1.077843423109	-1.547743089697	3.151361858627
C	-1.364499457359	0.421290237253	4.856213709747
H	0.429085642668	1.015559905915	3.975866380691
H	-1.309088098198	0.259402161986	1.410714804881
H	-2.186778951946	-0.286998178193	4.898757227258
H	-1.387903578635	1.241662995463	5.564424542122
H	2.908900493525	3.571036727959	-1.285994534441
H	1.139113378775	3.815174989703	-0.807314355963
H	0.349993101756	-2.208699837214	0.763839746492
H	-1.398522467956	-2.092074164687	0.740004484778

Ethylene

Energy: -78.620522 au

Energy including zero-point energy correction: -78.569519 au

Geometry:

C	-0.440231732906	-0.353413078080	-0.350094631351
C	0.440231648454	0.353413351652	0.350094459373
H	-0.167173408203	-0.848643233893	-1.275884097803
H	-1.469356082344	-0.465146837351	-0.025566462311
H	1.469356592509	0.465145211388	0.025567471316
H	0.167173903590	0.848641602493	1.275885136504

^{1}H, ^{13}C and 2D NMR Spectra

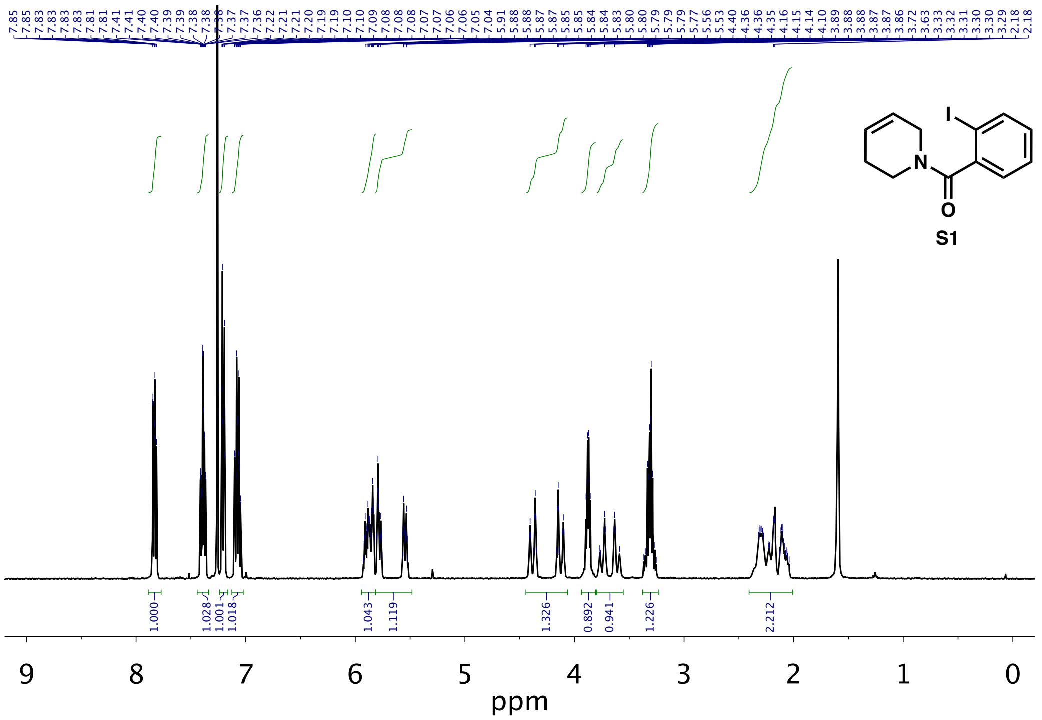

Figure S23. ^{1}H NMR (400 MHz, $CDCl_3$, 25 °C) of **S1**.

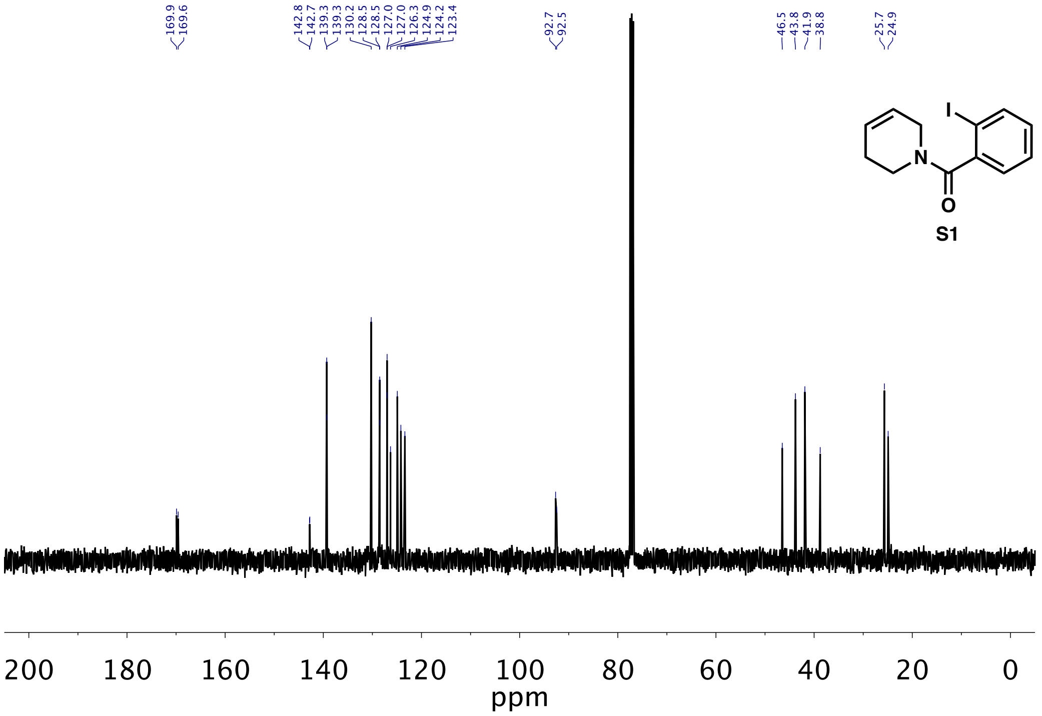

Figure S24. ^{13}C NMR (101 MHz, $CDCl_3$, 25 °C) of **S1**.

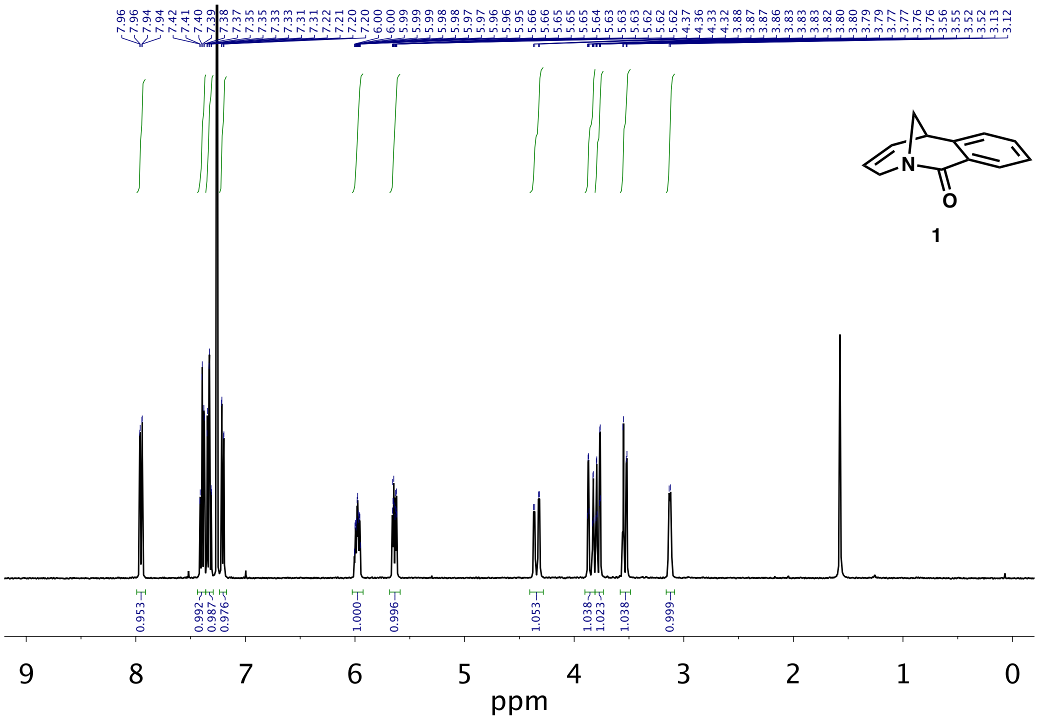

Figure S25. ^{1}H NMR (400 MHz, $CDCl_3$, 25 °C) of **1**.

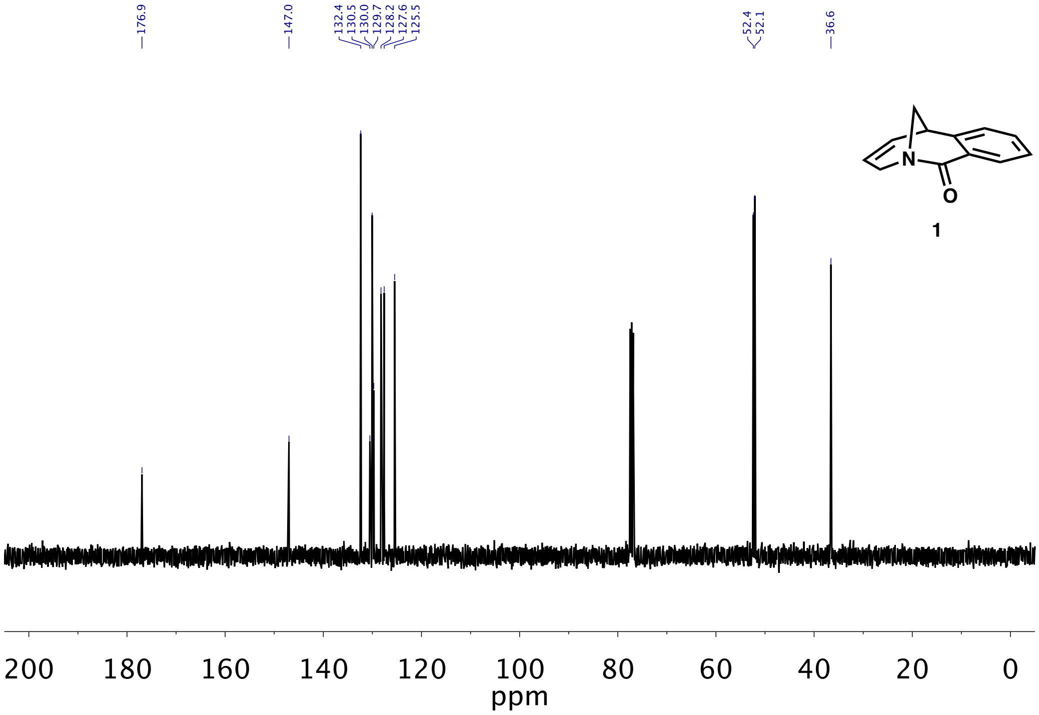

Figure S26. ^{13}C NMR (101 MHz, $CDCl_3$, 25 °C) of **1**.

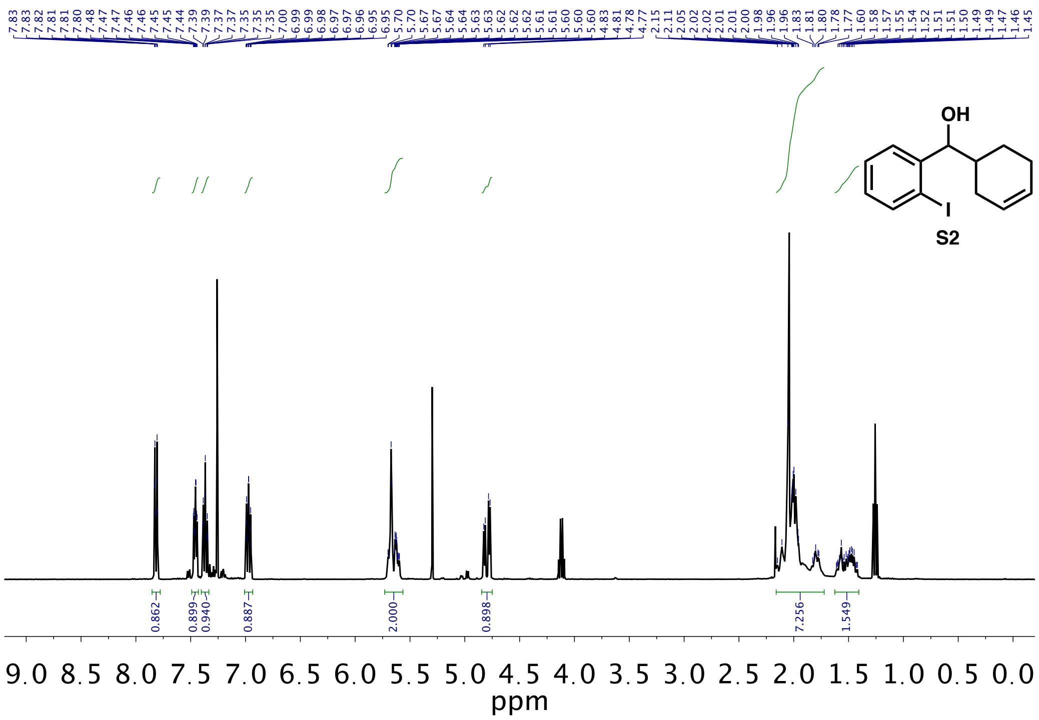

Figure S27. ^{1}H NMR (400 MHz, $CDCl_3$, 25 °C) of **S2**.

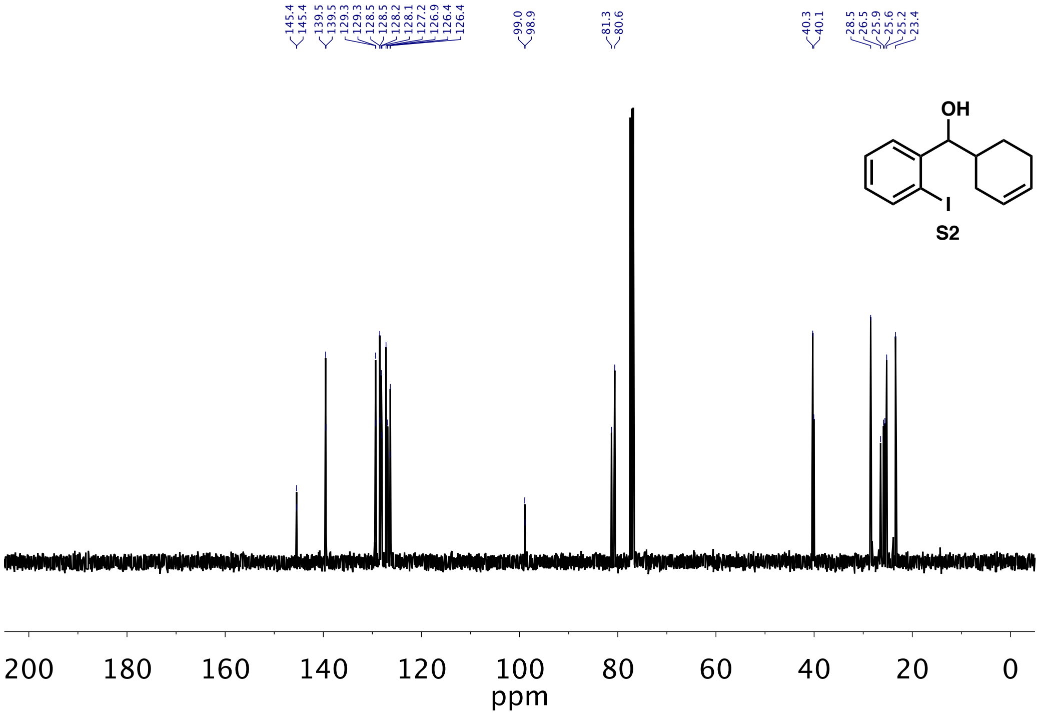

Figure S28. ^{13}C NMR (101 MHz, $CDCl_3$, 25 °C) of **S2**.

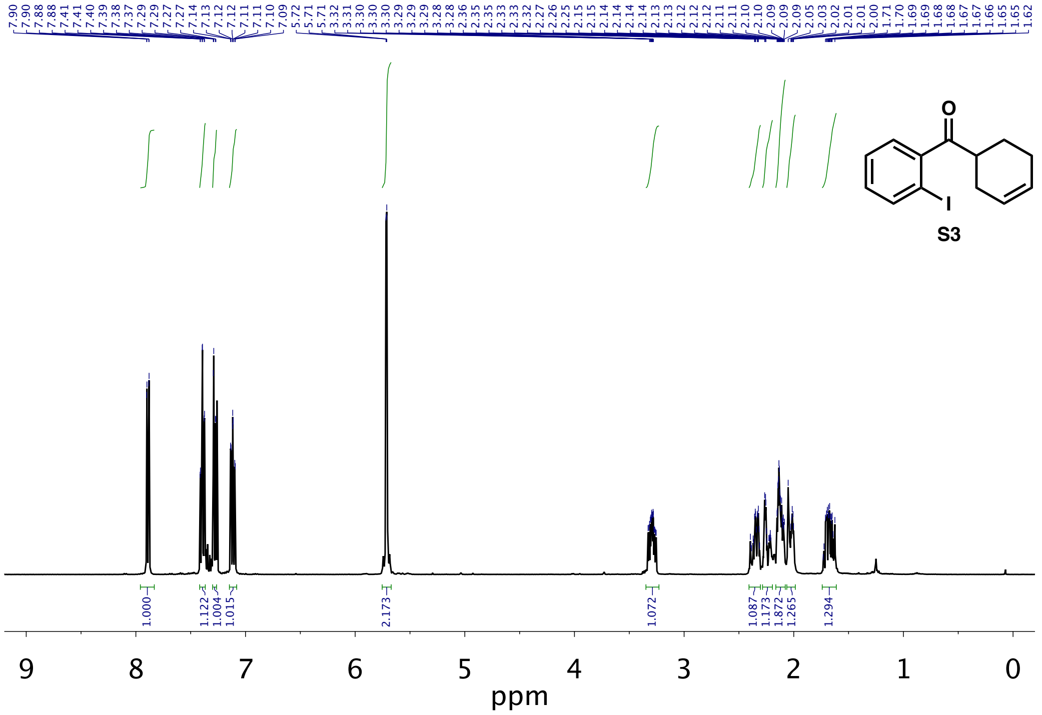

Figure S29. ^{1}H NMR (400 MHz, $CDCl_3$, 25 °C) of **S3**.

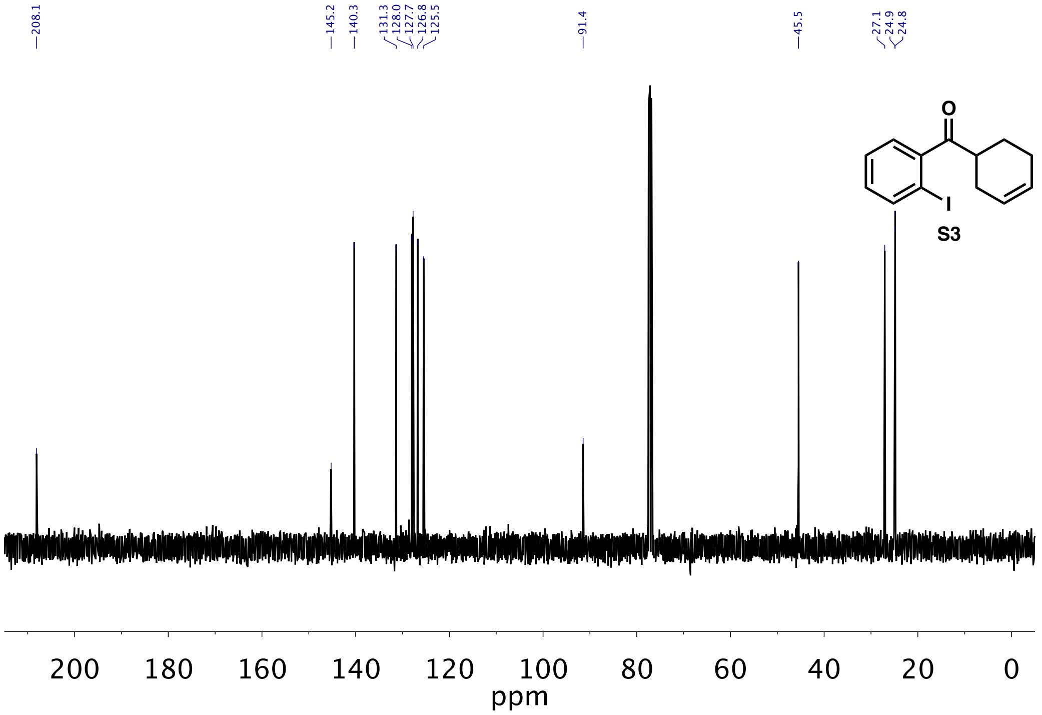

Figure S30. ^{13}C NMR (101 MHz, $CDCl_3$, 25 °C) of **S3**.

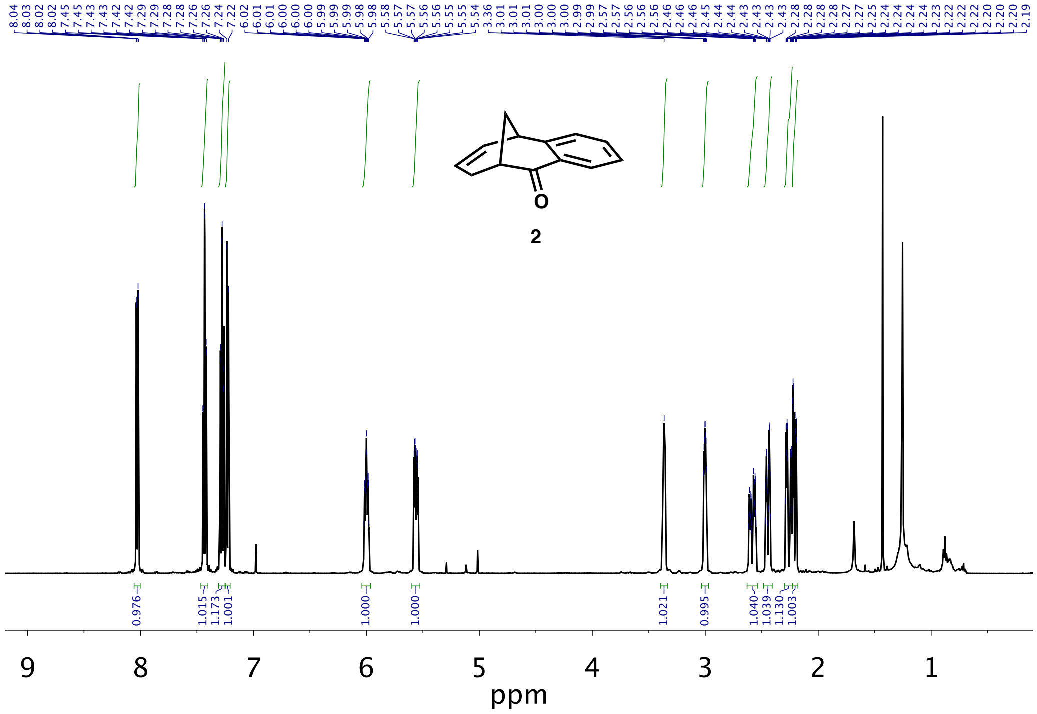

Figure S31. ^{1}H NMR (500 MHz, $CDCl_3$, 25 °C) of **2**.

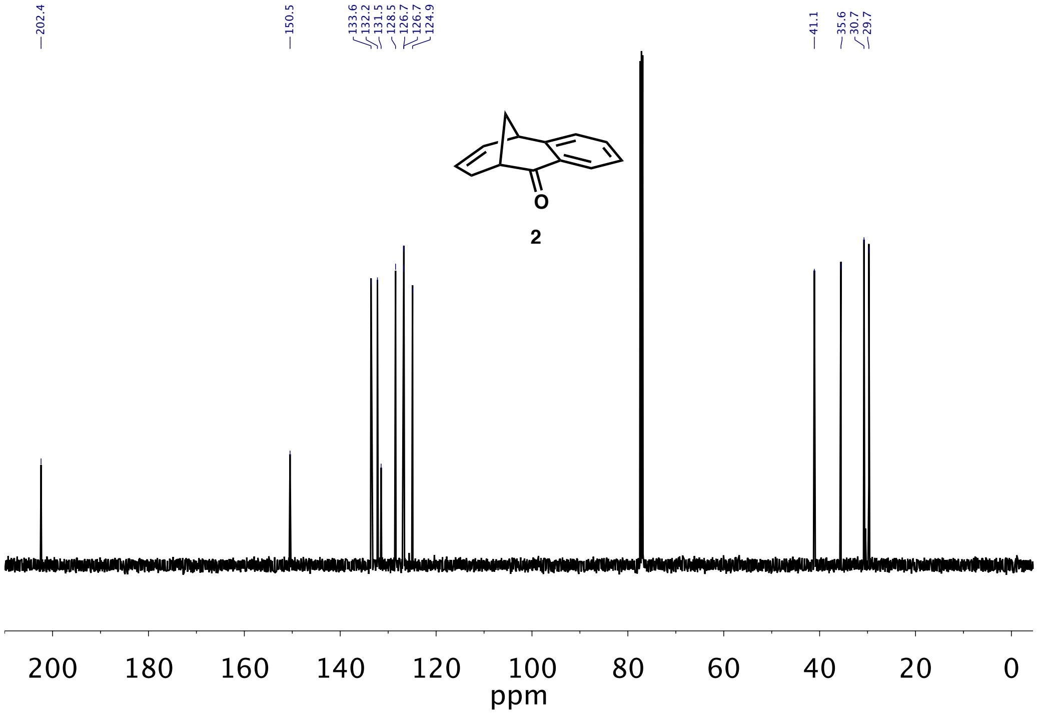

Figure S32. ^{13}C NMR (126 MHz, $CDCl_3$, 25 °C) of **2**.

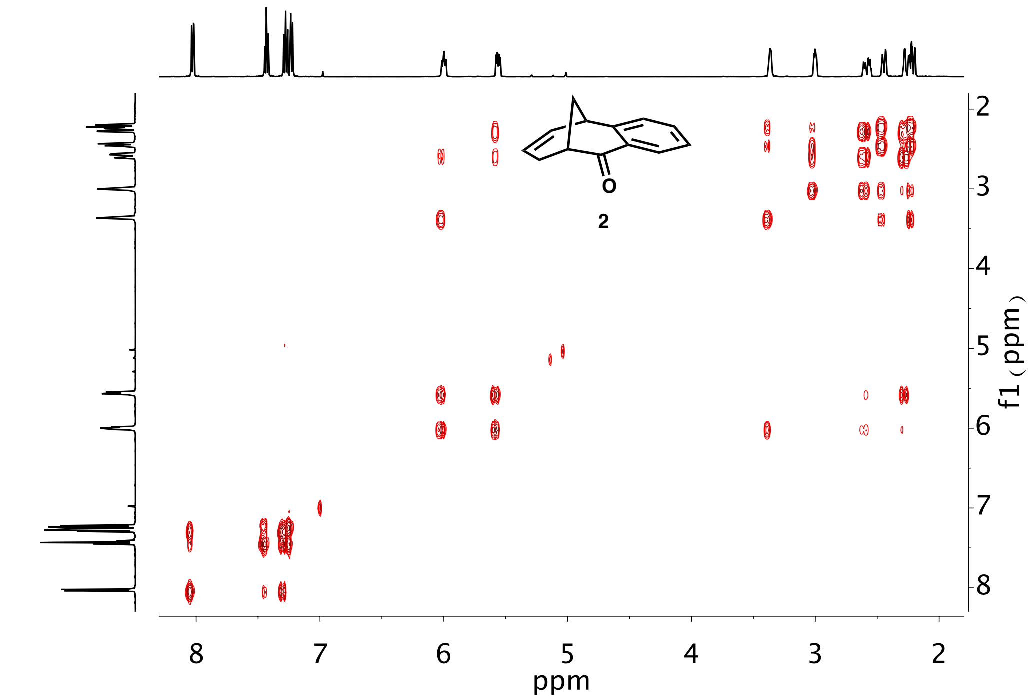

Figure S33. COSY (500 MHz, $CDCl_3$, 25 °C) of **2**.

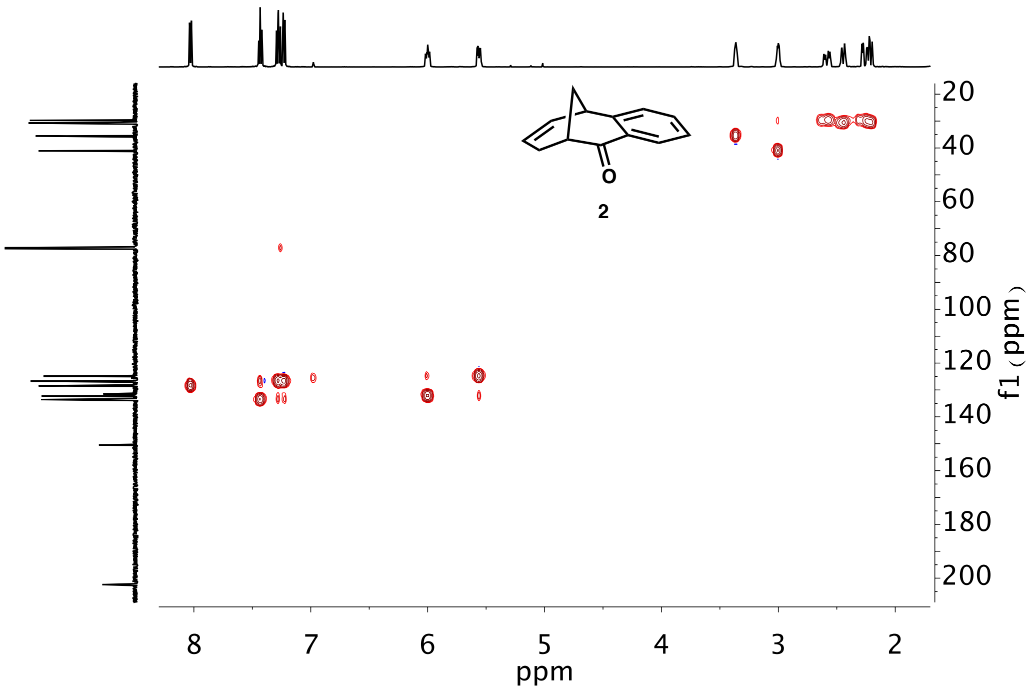

Figure S34. HSQC (500 MHz, $CDCl_3$, 25 °C) of **2**.

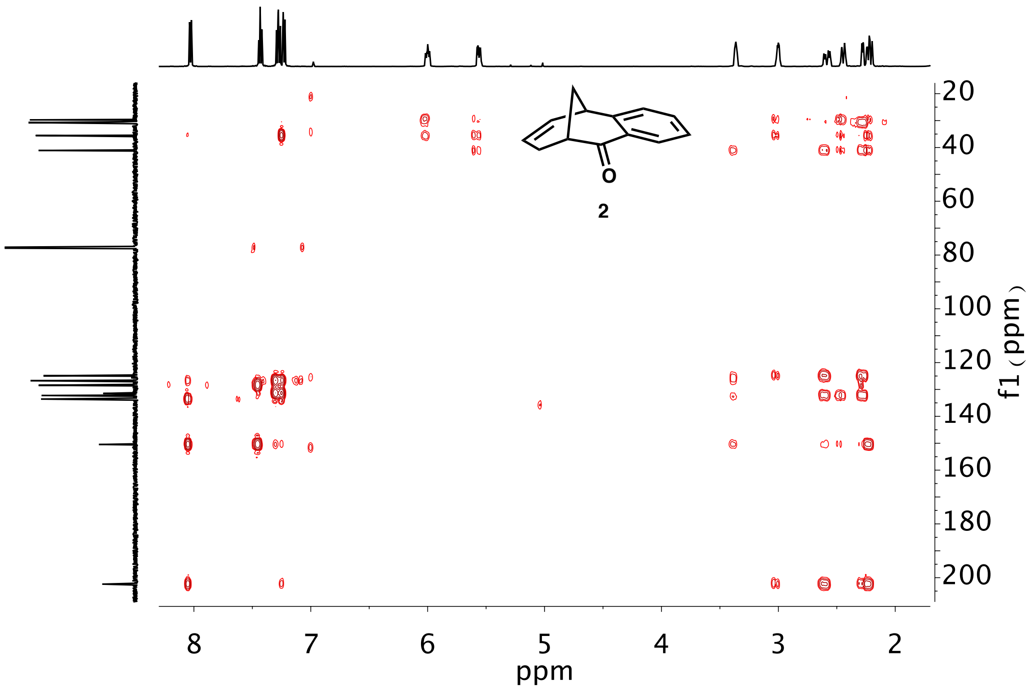

Figure S35. HMBC (500 MHz, CDCl₃, 25 °C) of **2**.

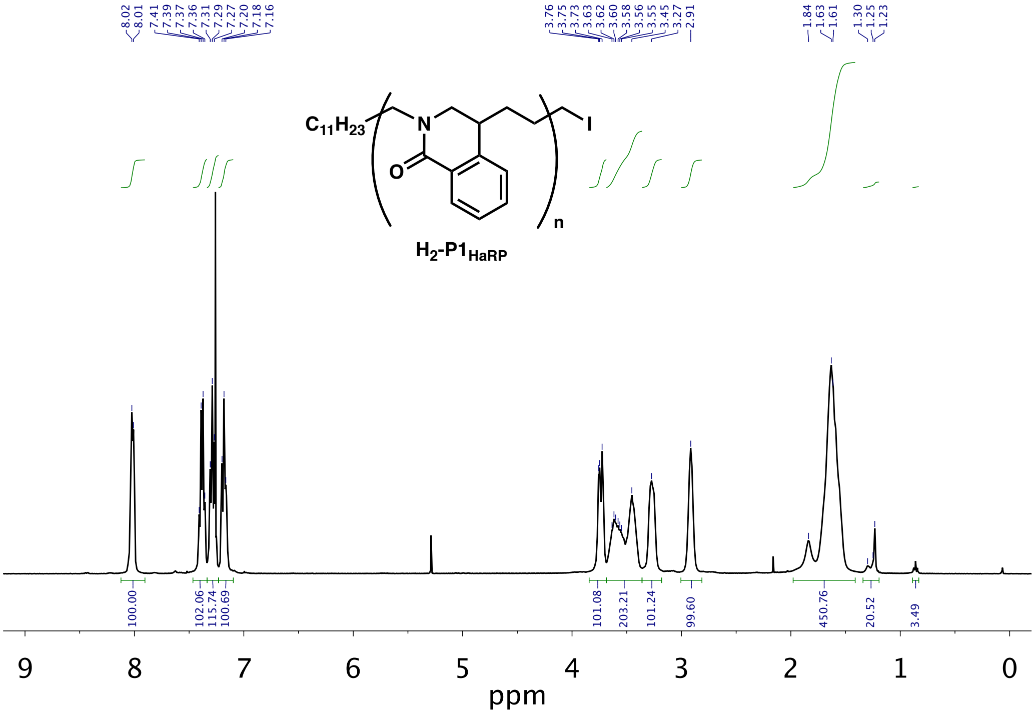

Figure S36. ^{1}H NMR (400 MHz, CDCl$_3$, 25 °C) of **H$_2$-P1$_{HaRP}$**.

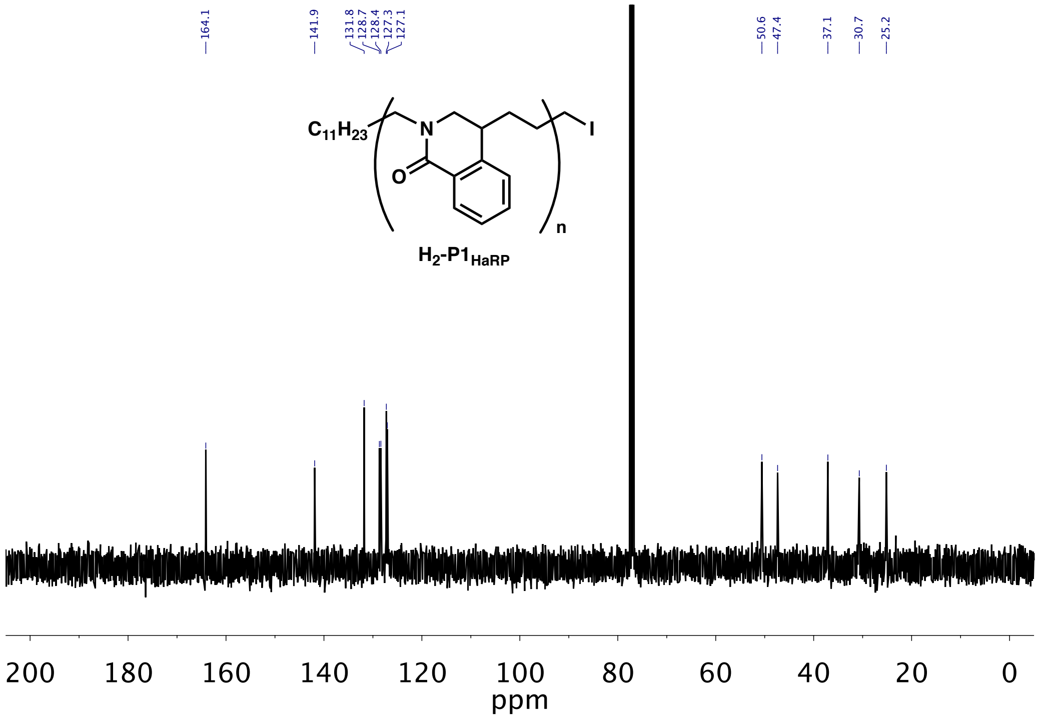

Figure S37. ${}^{13}C$ NMR (101 MHz, $CDCl_3$, 25 °C) of **H_2-P1$_{HaRP}$**.

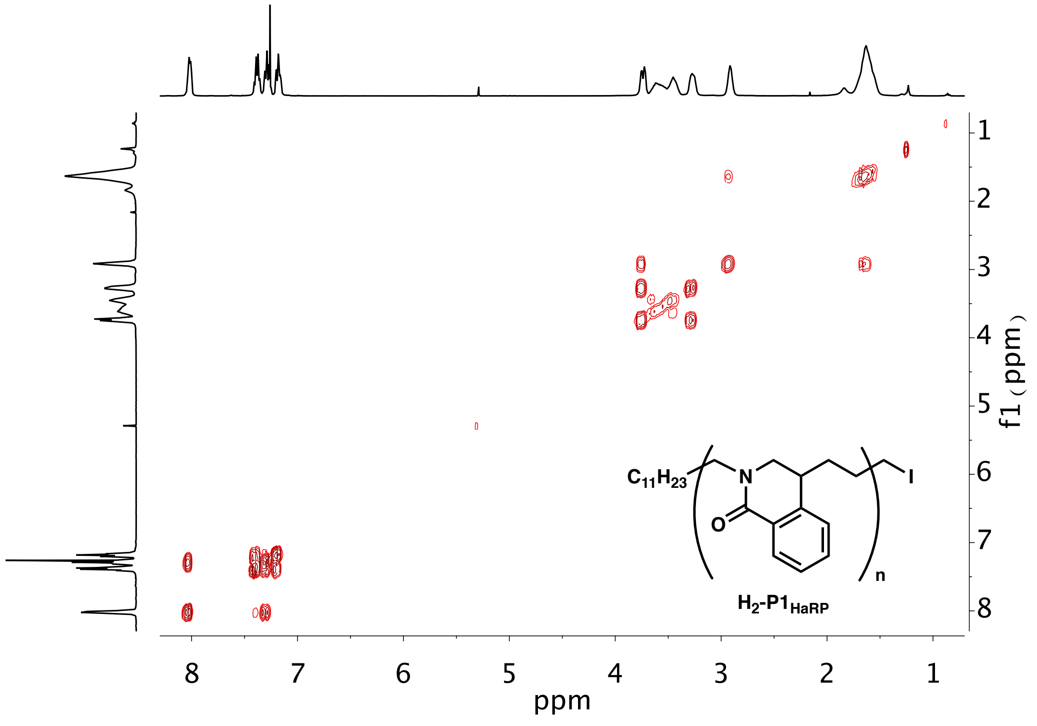

Figure S38. COSY (400 MHz, $CDCl_3$, 25 °C) of **H_2-P1$_{HaRP}$**.

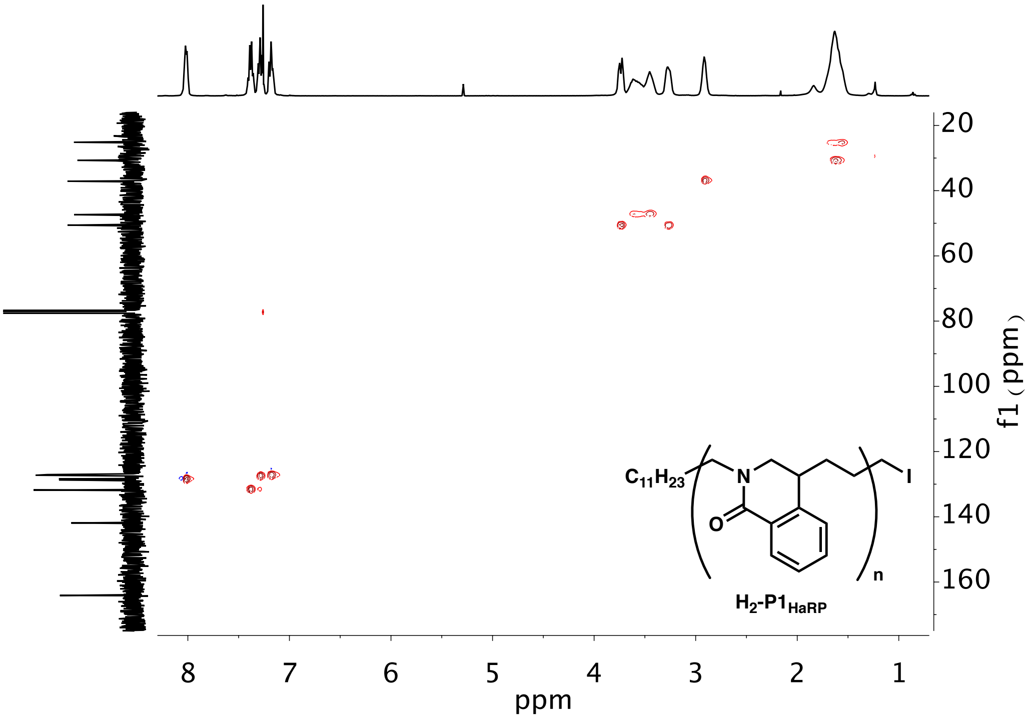

Figure S39. HSQC (400 MHz, $CDCl_3$, 25 °C) of **H_2-P1$_{HaRP}$**.

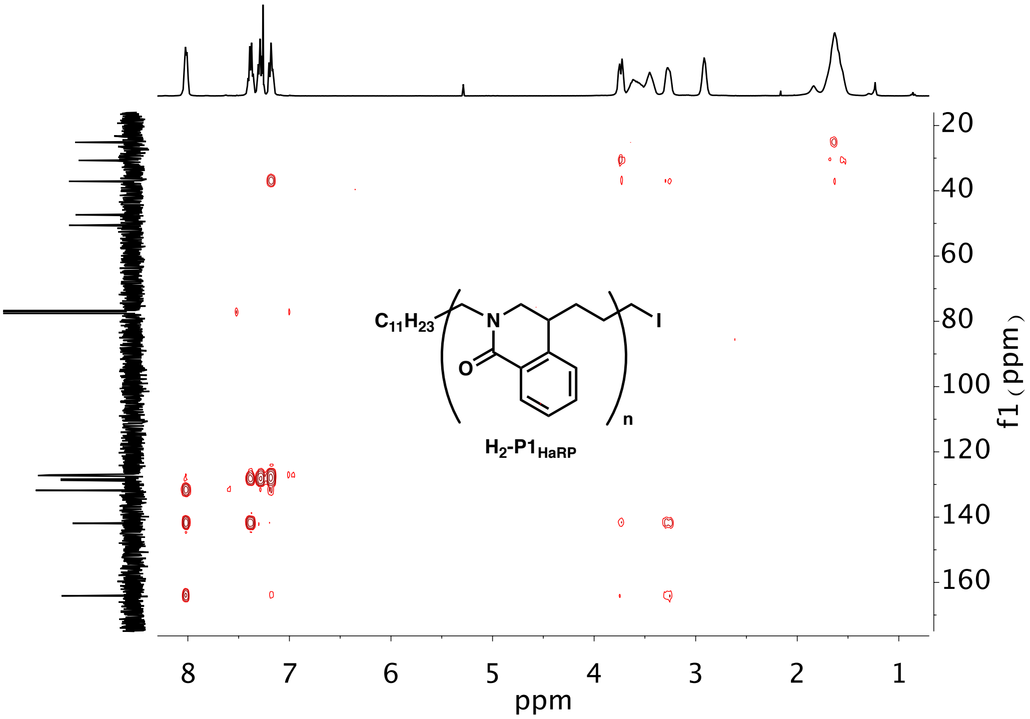

Figure S40. HMBC (400 MHz, $CDCl_3$, 25 °C) of **H_2-P1$_{HaRP}$**.

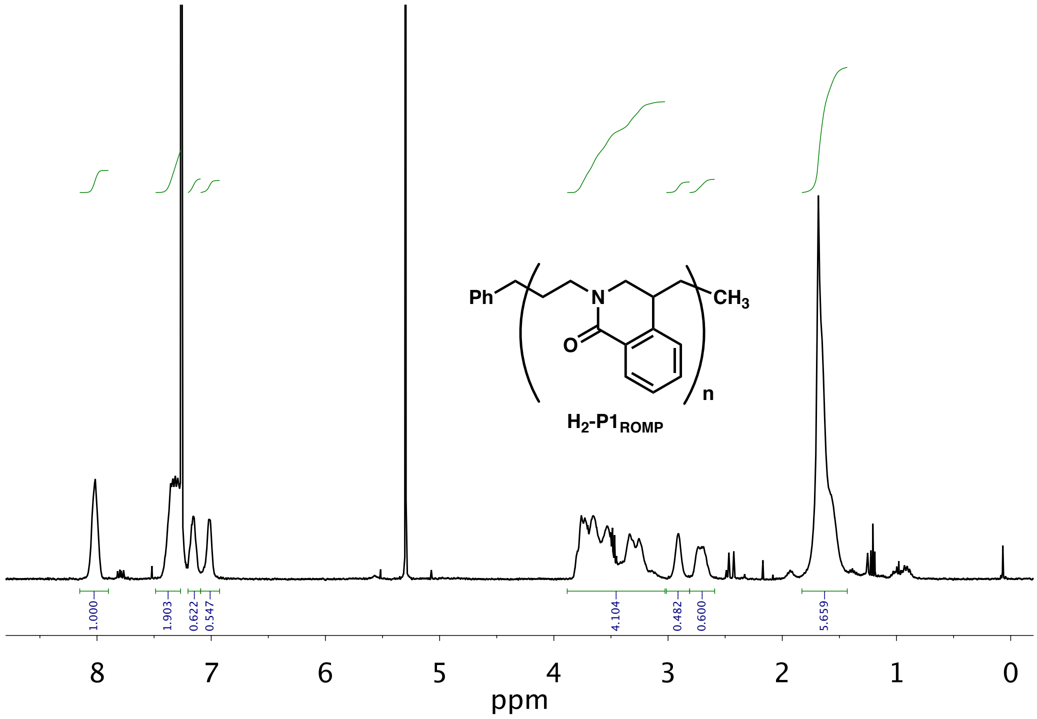

Figure S41. ^{1}H NMR (400 MHz, $CDCl_3$, 25 °C) of **H_2-P1$_{ROMP}$** (M_n: 4.5 kg/mol, *Đ*: 1.20).

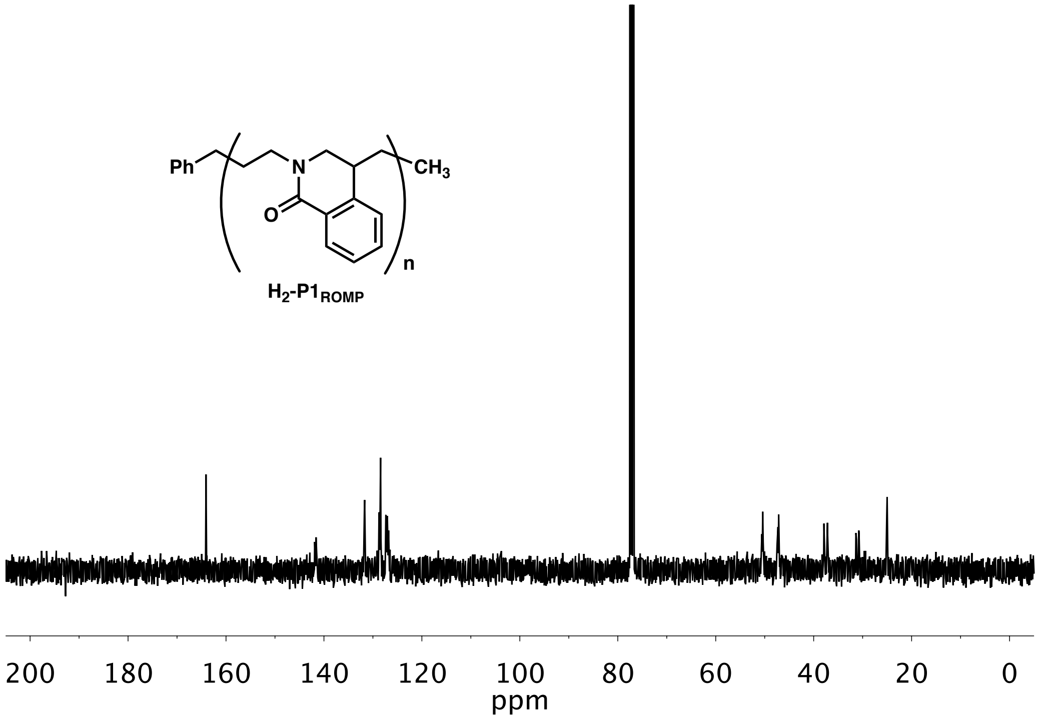

Figure S42. ^{13}C NMR (101 MHz, $CDCl_3$, 25 °C) of **H_2-P1$_{ROMP}$** (M_n: 4.5 kg/mol, *Đ*: 1.20).

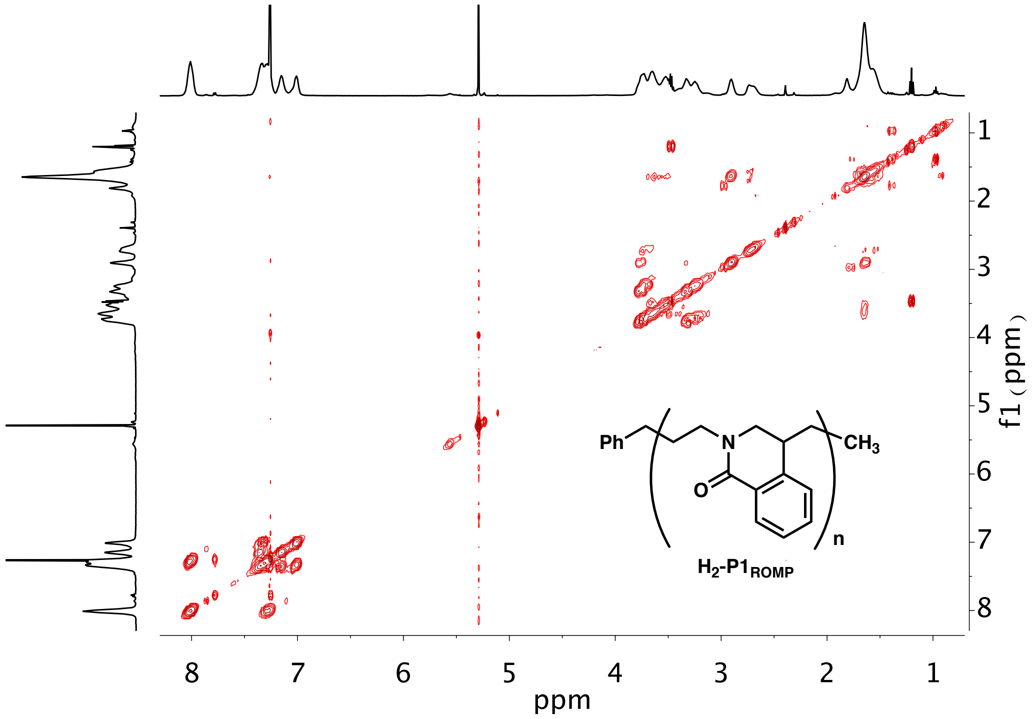

Figure S43. COSY (500 MHz, $CDCl_3$, 25 °C) of **H_2-P1$_{ROMP}$** (M_n: 9.3 kg/mol, *Đ*: 1.26).

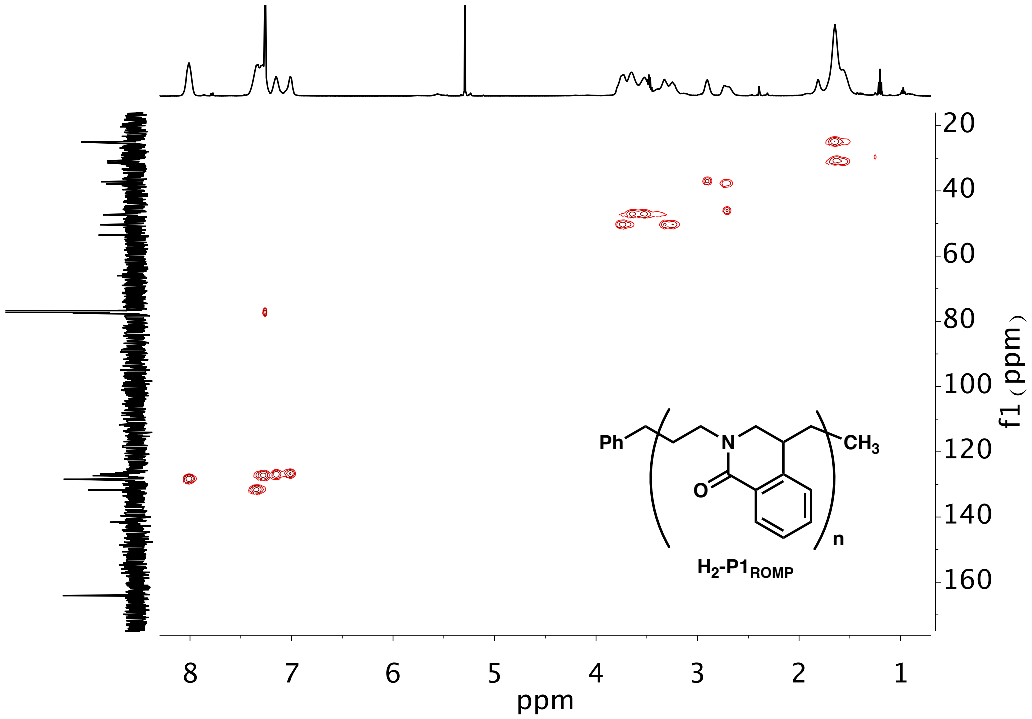

Figure S44. HSQC (500 MHz, $CDCl_3$, 25 °C) of **H_2-P1$_{ROMP}$** (M_n: 9.3 kg/mol, *Đ*: 1.26).

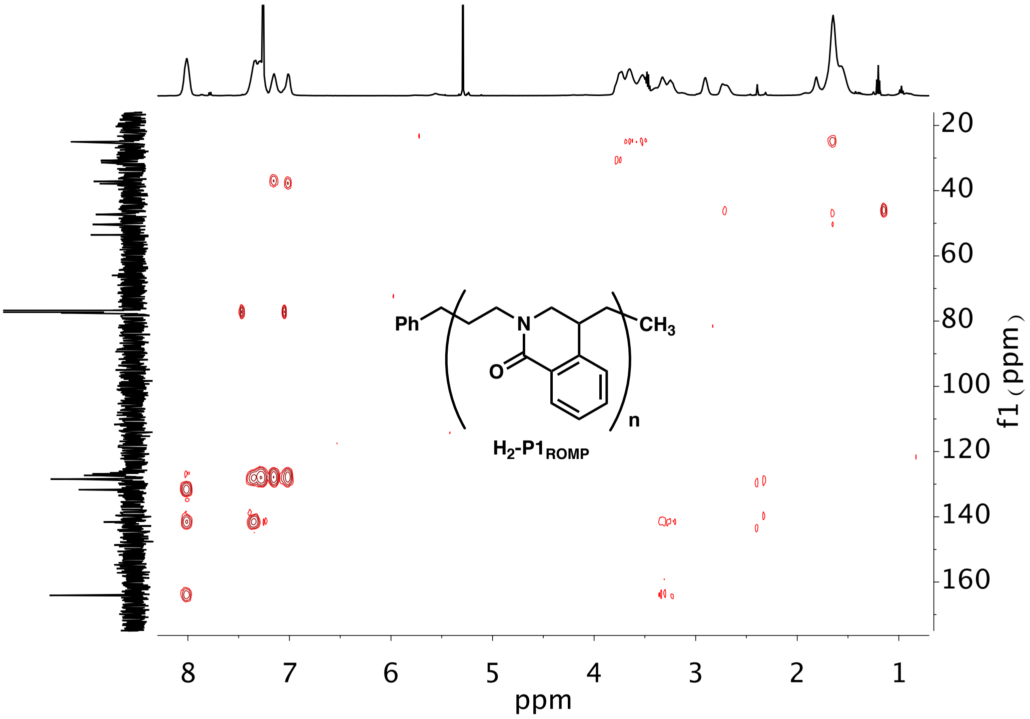

Figure S45. HMBC (500 MHz, $CDCl_3$, 25 °C) of **H_2-P1$_{ROMP}$** (M_n: 9.3 kg/mol, *Đ*: 1.26).

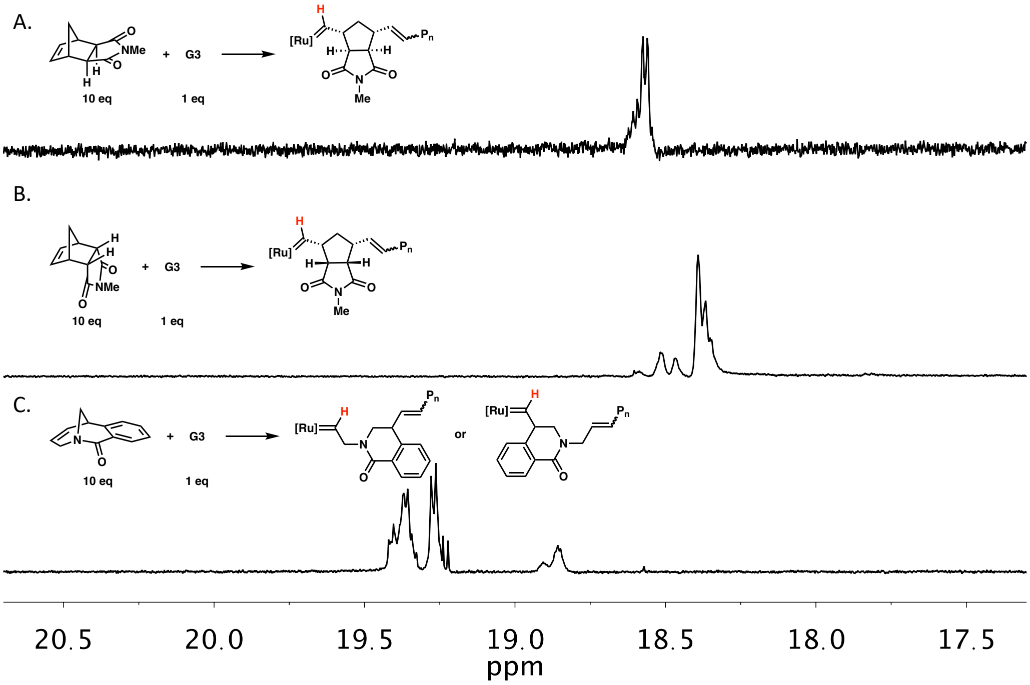

Figure S46. *In situ* ^{1}H NMR for propagating alkylidene chain-ends in ROMP of different monomers: (A) *exo*-methyl norbornene imide, (B) *endo*-methyl norbornene imide and (C) twisted amide **1**.

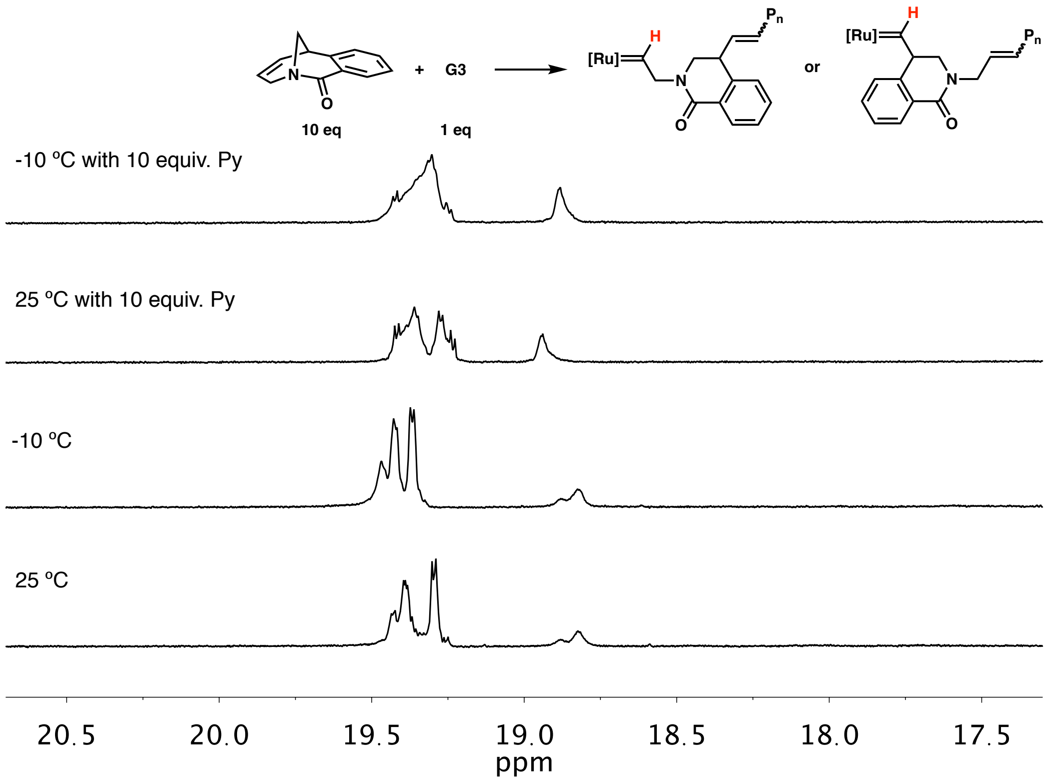

Figure S47. *In situ* ^{1}H NMR for propagating alkylidene chain-ends in ROMP of **1**.

References

1. J. A. Love, J. P. Morgan, T. M. Trnka and R. H. Grubbs, *Angew. Chem. Int. Ed.*, 2002, **41**, 4035-4037.
2. (a) F. Hu, P. Nareddy, R. Lalancette, F. Jordan and M. Szostak, *Org. Lett.*, 2017, **19**, 2386-2389; (b) F. Hu, R. Lalancette and M. Szostak, *Angew. Chem. Int. Ed.*, 2016, **55**, 5062-5066; (c) R. Grigg, V. Santhakumar, V. Sridharan, P. Stevenson, A. Teasdale, M. Thornton-Pett and T. Worakun, *Tetrahedron*, 1991, **47**, 9703-9720; (d) R. Grigg, V. Sridharan, P. Stevenson and T. Worakun, *J. Chem. Soc., Chem. Commun.*, 1986, 1697-1699.
3. (a) J. Cvengroš, D. Stolz and A. Togni, *Synthesis*, 2009, 2818-2824; (b) M. A. Schade, S. Yamada and P. Knochel, *Chem. Eur. J.*, 2011, **17**, 4232-4237.
4. M. M. Abelman, T. Oh and L. E. Overman, *J. Org. Chem.*, 1987, **52**, 4130-4133.
5. L. Fu, M. Xu, J. Yu and W. R. Gutekunst, *J. Am. Chem. Soc.*, 2019, **141**, 2906-2910.
6. S. Kobayashi, C. Lu, T. R. Hoye and M. A. Hillmyer, *J. Am. Chem. Soc.*, 2009, **131**, 7960-7961.
7. R. M. Parrish, L. A. Burns, D. G. A. Smith, A. C. Simmonett, A. E. DePrince, E. G. Hohenstein, U. Bozkaya, A. Y. Sokolov, R. Di Remigio, R. M. Richard, J. F. Gonthier, A. M. James, H. R. McAlexander, A. Kumar, M. Saitow, X. Wang, B. P. Pritchard, P. Verma, H. F. Schaefer, K. Patkowski, R. A. King, E. F. Valeev, F. A. Evangelista, J. M. Turney, T. D. Crawford and C. D. Sherrill, *J. Chem. Theory Comput.*, 2017, **13**, 3185-3197.
8. (a) R. Szostak and M. Szostak, *J. Org. Chem.*, 2019, **84**, 1510-1516; (b) R. Szostak, J. Aubé and M. Szostak, *J. Org. Chem.*, 2015, **80**, 7905-7927; (c) A. Greenberg, D. T. Moore and T. D. DuBois, *J. Am. Chem. Soc.*, 1996, **118**, 8658-8668; (d) A. Greenberg and C. A. Venanzi, *J. Am. Chem. Soc.*, 1993, **115**, 6951-6957.
